# Senescence-Related SOCS1, SOCS2, and GADD45G Identify an Immune-Associated Molecular Signature in Metabolic Dysfunction-Associated Steatotic Liver Disease

**DOI:** 10.3390/biom16081154

**Published:** 2026-08-07

**Authors:** Wenjing Tang, Weixia Wang, Shuyang Zhang, Xin Zhou, Linfeng He, Tianshu Zeng

**Affiliations:** 1 Department of Endocrinology, Union Hospital, Tongji Medical College, Huazhong University of Science and Technology, Wuhan 430022, Hubei, China; d202482188@hust.edu.cn (W.T.); u201810265@hust.edu.cn (W.W.); shuyangzhang@hust.edu.cn (S.Z.); vgirlxin@hust.edu.cn (X.Z.); 2 Diabetes and Metabolic Disease Clinical Research Center of Hubei Province, Wuhan 430022, Hubei, China; 3 Hubei Key Laboratory of Metabolic Abnormalities and Vascular Aging, Wuhan 430022, Hubei, China; 4 Hubei Branch of National Center for Clinical Medical Research of Metabolic Diseases, Wuhan 430022, Hubei, China

**Keywords:** metabolic dysfunction-associated steatotic liver disease, cellular senescence, immune infiltration, GADD45G, SOCS1, SOCS2

## Abstract

Senescence-related molecular nodes linking hepatic stress with immune dysregulation in metabolic dysfunction-associated steatotic liver disease (MASLD) remain poorly defined. This study aimed to identify and experimentally validate a senescence-related gene (SRG) signature associated with immune dysregulation in MASLD. Four transcriptomic datasets (127 controls, 127 MASLD) were integrated as the discovery cohort. Candidate SRGs were identified through differential expression analysis, weighted gene co-expression network analysis, and three machine learning algorithms. Immune infiltration was assessed using CIBERSORT, and a three-gene signature was evaluated in internal and external cohorts. Potential GADD45G-associated compounds were predicted by molecular docking. Experimental validation was performed in high-fat diet (HFD)-induced obese mice and palmitic acid (PA)-stimulated bone marrow-derived macrophages (BMDMs). Single-cell RNA sequencing analysis of GSE300744 was further performed to evaluate GADD45G distribution in hepatic cell populations. SOCS1, SOCS2, and GADD45G were identified as core SRGs. Their expression was reduced in MASLD liver tissues and correlated with histological features and immune alterations. The three-gene signature showed favorable classification performance in validation cohorts. In HFD mice, GADD45G expression was decreased at both mRNA and protein levels, with reduced immunoreactivity in F4/80-positive hepatic macrophages. Single-cell analysis further supported macrophage-associated expression of Gadd45g. In PA-stimulated BMDMs, reduced GADD45G protein expression was partially restored by camptothecin and myristicin treatment. Overall, SOCS1, SOCS2, and GADD45G represent a candidate senescence-related immune signature associated with MASLD. The macrophage-associated reduction in GADD45G and its modulation in vitro provide preliminary evidence for a potential role of GADD45G in MASLD-associated immune dysregulation.

## 1. Introduction

Metabolic dysfunction-associated steatotic liver disease (MASLD) is the most common chronic liver disease worldwide and is closely linked to obesity, insulin resistance, and type 2 diabetes [1,2,3]. MASLD substantially increases the risk of liver-related complications and adverse cardiometabolic outcomes [2,3]. Although pharmacological options have begun to emerge for selected patients, disease-modifying strategies remain limited, and the molecular mechanisms that drive disease progression are not fully understood. Identification of disease-relevant molecular regulators is therefore important for improving risk stratification and developing targeted interventions.

Cellular senescence has increasingly been recognized as an important pathogenic process in MASLD, especially during the transition from simple steatosis to steatohepatitis and fibrosis [4]. Senescence is a state of stable cell-cycle arrest induced by stressors such as metabolic overload, lipotoxicity, oxidative stress, and DNA damage [4,5]. Senescent hepatocytes and other hepatic cells can acquire a senescence-associated secretory phenotype (SASP), characterized by the release of cytokines, chemokines, and matrix-remodeling factors [6]. These mediators reshape the hepatic microenvironment, sustain local inflammation, and promote fibrogenic activation [6,7,8].

Persistent metabolic stress also promotes hepatocyte injury and the release of damage-associated molecular patterns (DAMPs), which act as danger signals within the liver. Together with SASP components, these signals recruit and activate innate and adaptive immune cells, including macrophages, T lymphocytes, and natural killer cells [4,7,9]. A key feature of this process is the shift of liver-resident Kupffer cells and recruited monocyte-derived macrophages from a homeostatic state toward pro-inflammatory phenotypes [10]. This immune remodeling amplifies hepatocellular injury and metabolic dysfunction, creating a feed-forward cycle of inflammation and tissue damage [10,11].

Cellular senescence and immune activation are therefore closely interconnected in MASLD [12,13]. SASP factors help shape the inflammatory infiltrate, whereas activated immune cells can further induce or reinforce senescence programs in neighboring liver cells [8]. However, the molecular regulators that coordinate this senescence–immune interaction in MASLD remain insufficiently characterized. Defining these regulators may provide biomarkers and mechanistic clues for future therapeutic development.

High-throughput transcriptomic profiling and computational biology provide useful approaches for identifying candidate regulatory genes from large datasets. Integrative bioinformatics combined with machine learning can prioritize disease-associated genes, but experimental validation is required to support their biological relevance [14,15].

In this study, we integrated multiple liver transcriptomic datasets and applied weighted gene co-expression network analysis, machine learning approaches, immune infiltration analysis, single-cell RNA sequencing validation, and experimental validation to identify senescence-related genes associated with MASLD. We focused on the relationship between core senescence-related genes (SRGs) and immune alterations. We further explored small molecules associated with GADD45G regulation. Our findings suggest that SOCS1, SOCS2, and GADD45G constitute a candidate senescence-related immune-associated signature in MASLD, with GADD45G showing macrophage-associated expression patterns and potential involvement in MASLD-related immune dysregulation.

## 2. Materials and Methods

### 2.1. Data Download and Processing

Human liver transcriptomic datasets related to metabolic dysfunction-associated steatotic liver disease (MASLD) were obtained from the NCBI Gene Expression Omnibus (GEO) database (https://www.ncbi.nlm.nih.gov/geo/; accessed on 1 May 2025). The inclusion criteria were as follows: (1) samples were derived from human liver tissues; (2) datasets included samples with clearly defined MASLD diagnosis, with or without healthy control groups; (3) mRNA expression profiles were available (microarray or RNA-seq); and (4) relevant clinical information, such as age, body mass index (BMI), and liver fibrosis score, was available for downstream clinical correlation analyses. Based on these criteria, four datasets—GSE48452 [16], GSE89632 [17], GSE61260 [18], and GSE96971 [19]—were included in the discovery cohort, comprising a total of 127 healthy controls and 127 MASLD samples. Table 1 provides a comprehensive summary of these datasets. Shared genes were aligned across datasets, negative values were replaced with zero, and batch effects were removed using the “sva” package (version 3.60.0) [20]. Boxplots were created pre- and post-correction to evaluate sample distribution consistency and confirm effective data integration. The GSE135251 [21] dataset served as the external validation dataset in this investigation. All computational analyses were implemented in R software (version 4.4.3).

### 2.2. Differentially Expressed Gene (DEG) Identification in MASLD

Differential expression analysis was performed on the integrated expression matrix after batch correction. MASLD samples from all included datasets were compared with the available healthy control samples in the merged cohort using the “limma” package (version 3.68.3) in R [22]. Genes were classified as differentially expressed if they exhibited a |log2 fold change| (|log2 FC|) greater than 0.5 and an adjusted *p*-value less than 0.05 [23]. Considering the biological heterogeneity of human MASLD liver tissues, this moderate fold-change threshold was applied to retain biologically relevant candidates with moderate, but consistent expression alterations for subsequent integrative analyses [23,24]. Heatmaps and volcano plots were created using the “pheatmap” (version 1.0.13) and “ggplot2” (version 4.0.3) packages in R to visualize the identified DEGs.

### 2.3. Weighted Gene Co-Expression Network Analysis (WGCNA)

WGCNA was performed using the R package “WGCNA” (version 1.74) [25] to identify gene modules associated with MASLD. The analysis was conducted on the integrated expression matrix after batch effect correction to improve network robustness. A scale-free co-expression network was developed using a soft-thresholding power of β = 3. Genes were clustered hierarchically using topological overlap, and modules were identified using a dynamic tree-cutting algorithm with a minimum module size of 30 and a deep split setting of 2. Modules exhibiting similar expression patterns were combined using a cutoff height of 0.25, corresponding to a module similarity greater than 0.75. The relationships between module eigengenes and clinical traits were evaluated to identify modules most significantly associated with MASLD. Hub genes within the key modules were identified based on module membership (MM) and gene significance (GS), reflecting the correlation between gene expression and clinical traits.

### 2.4. Acquisition of SRGs and Screening of Key SRGs

Human SRGs were obtained from the CellAge [26] and GenAge [27] databases, yielding a total of 1061 genes (Supplementary Table S1). Candidate SRGs associated with MASLD were identified by intersecting 1061 SRGs with DEGs and WGCNA-derived key module genes. Three machine learning algorithms were applied with a fixed random seed (seed = 42) to ensure reproducibility. LASSO logistic regression was performed using the glmnet package (version 5.0) [28] with 10-fold cross-validation under the binomial model, and genes with non-zero coefficients were retained. SVM-RFE was performed using the e1071 package (version 1.7.17) [29] with a linear kernel (*C* = 1) and 5-fold cross-validation. Random forest analysis was conducted using the randomForest package (version 4.7.1.2) [30] with 1000 trees, and feature importance was ranked using mean decrease Gini. Cross-validation, L1 regularization in LASSO, and out-of-bag (OOB) error estimation in random forest were applied to reduce the risk of overfitting. Core SRGs were defined as genes consistently identified by all three approaches.

### 2.5. Functional Enrichment and Immune Infiltration Analysis

The Gene Ontology (GO) resource (http://geneontology.org; accessed on 1 May 2025) offers structured, computable insights into gene and gene product functions. GO function enrichment encompasses three categories: molecular function (MF), biological process (BP), and cellular component (CC). The Kyoto Encyclopedia of Genes and Genomes (KEGG, http://www.genome.ad.jp/kegg/; accessed on 1 May 2025) is a comprehensive database that compiles genomic, chemical, and systemic functional data on biological systems. The R package “clusterProfiler” (version 4.20.0) [31] was used to conduct and visualize GO and KEGG pathway enrichment analyses. Immune cell infiltration was estimated using the CIBERSORT algorithm (R script version 1.03) [32] based on the LM22 leukocyte signature matrix with 1000 permutations. The relative proportions of 22 immune cell types were calculated, and Spearman correlation analysis was performed to evaluate the associations between core SRGs and immune cell infiltration.

### 2.6. Construction of Nomograms and Evaluation of the SRG-Based Molecular Signature

The classification performance of the identified genes was assessed using the “rms” package (version 8.1.1) [33] to construct a nomogram, and its accuracy was evaluated through a calibration curve. In the nomogram, each gene was assigned a score, and the cumulative score of the three genes was utilized to predict MASLD risk.

### 2.7. Validation of the Three-Gene Molecular Signature

The integrated dataset was randomly split into a training set and an internal validation set with a 7:3 ratio. A logistic regression model was constructed based on the core SRGs. The “pROC” package (version 1.19.0.1) was utilized to conduct ROC curve analysis [34], and the area under the curve (AUC) was calculated. The independent GSE135251 dataset was used for external validation. To further assess the robustness of the molecular signature, precision–recall (PR) curve analysis was performed using the “PRROC” package (version 1.4). In addition, sensitivity, specificity, and 95% confidence intervals (CIs) of the AUC were calculated to provide a comprehensive evaluation of model performance.

### 2.8. Evaluation of Dataset Integration Robustness

To evaluate whether the inclusion of GSE96971, which contained MASLD samples without matched healthy controls, affected the robustness of the integrated transcriptomic analysis, we repeated the differential expression analysis after excluding this dataset using the same preprocessing procedures and criteria (|log2FC| > 0.5 and adjusted *p*-value < 0.05). The consistency of gene-level log2 fold changes between the original and reanalyzed datasets was assessed using Spearman correlation analysis.

### 2.9. Correlation Between SRGs and Clinical Traits

To explore associations between three key SRGs and MASLD clinical pathological traits, Spearman correlation analysis was performed using clinical data from GEO datasets (GSE48452, GSE89632, GSE96971, GSE61260). Seven indicators were extracted: ballooning score, fibrosis stage, inflammation score, NAS, steatosis grade, BMI, and age.

### 2.10. Single-Cell RNA Sequencing Data Processing and Analysis

To validate the cell expression patterns of candidate SRGs, the single-cell RNA-seq dataset GSE300744 [35], comprising liver tissues from HFD-fed and control mice, was retrieved from the GEO database. Data preprocessing and quality control were performed using the Seurat package (version 5.0.0). Cells with fewer than 500 or more than 6000 detected genes (nFeature_RNA), fewer than 1000 or more than 30,000 total UMI counts (nCount_RNA), or mitochondrial gene content exceeding 15% were excluded. Potential doublets were identified using DoubletFinder (version 2.0.3) and removed before downstream analysis. High-quality cells were normalized using NormalizeData function implemented in the Seurat package (version 5.0.0), followed by dimensionality reduction and batch correction using Harmony (version 2.0.1). Graph-based clustering was performed based on the top 20 principal components, and UMAP was used for visualization. Major liver cell populations were annotated according to canonical marker genes. The expression patterns of SOCS1, SOCS2, and GADD45G across different cell populations were visualized using dot plots.

### 2.11. Animal Experiment

Male C57BL/6J wild-type mice were obtained from Wuhan Youdu Biotechnology (Wuhan, China). All mice were kept in a controlled environment with a consistent light/dark cycle (light from 06:00 to 18:00, dark from 18:00 to 06:00) and a stable temperature of 22 ± 2 °C, with unrestricted access to food and water. This study utilized male mice aged 7 to 8 weeks. Mice were housed in groups for one week prior to the experiment and then fed a high-fat diet (60% of calories from fat) for 16 weeks. At the end of the experimental period, mice were deeply anesthetized with 4% isoflurane. Euthanasia was then performed by cervical dislocation, ensuring the absence of vital signs before tissue collection. All animal procedures were approved by the Institutional Animal Care and Use Committee (IACUC) of Shouzheng Pharma (Wuhan, China) Biotechnology Co., Ltd. and conducted in accordance with the relevant animal welfare guidelines (approval SZHY-IACUC-No.2025112101).

### 2.12. Hematoxylin-and-Eosin Staining of Mouse Liver Tissues

Paraffin-embedded mouse liver sections (5 μm) were deparaffinized in xylene, rehydrated through graded ethanol, and rinsed with distilled water. Sections were stained with hematoxylin for 5 min, differentiated with 1% hydrochloric acid ethanol for 30 s, and rinsed with running water to blue the nuclei. After eosin staining for 2 min, sections were dehydrated via graded ethanol, cleared in xylene, and mounted with neutral balsam. Images were captured at 200× magnification using a light microscope. Hepatic steatosis, inflammation, and hepatocellular ballooning were evaluated according to the MASLD Activity Score (NAS) criteria [36]. Three random fields per section and three biological replicates per group were analyzed.

### 2.13. Immunohistochemical (IHC) Staining of Mouse Liver Tissues

Liver tissues from normal diet (ND)- and HFD-fed mice were fixed in 4% paraformaldehyde, dehydrated, paraffin-embedded, and sectioned into 5 μm slices. After deparaffinization, antigen retrieval was performed in citrate buffer (pH 6.0) via pressure cooking. Endogenous peroxidase was blocked with 3% H_2_O_2_ and non-specific binding with 5% BSA. Sections were incubated overnight at 4 °C with primary antibodies against GADD45G (1:200, Invitrogen, Carlsbad, CA, USA), SOCS1 (1:500, Invitrogen, Carlsbad, CA, USA), and SOCS2 (1:200, Invitrogen, Carlsbad, CA, USA), followed by HRP-conjugated secondary antibody (1:500, Invitrogen, Carlsbad, CA, USA) at 37 °C for 30 min. Staining was visualized with DAB chromogen, counterstained with hematoxylin, dehydrated, and mounted. Images were captured at 200× magnification, and positive expression was quantified using ImageJ software (version 1.53). Three fields per section and three replicates per group were analyzed.

### 2.14. Quantitative Real-Time PCR

Total RNA was extracted from cells or freshly harvested liver tissue using TRIzol reagent and further purified with the RNAeasy Mini Kit (Vazyme, Nanjing, China) according to the manufacturer’s instructions. To eliminate potential genomic DNA contamination, RNA samples were treated with DNase (Vazyme, Nanjing, China). RNA concentration and purity were assessed by an ultramicro spectrophotometer (KAIAO, Beijing, China). First-strand cDNA synthesis was performed using the SuperScript First-Strand Synthesis System (Thermo Fisher Scientific, Waltham, MA, USA). Quantitative real-time PCR (qPCR) was carried out using SYBR Green master mix (Vazyme, Nanjing, China) on a CFX real-time PCR detection system (Bio-Rad, Hercules, CA, USA). Gene expression levels were normalized using β-actin as an internal control, and the primer sequences used in this study are listed in Table 2.

### 2.15. Immunofluorescence Staining

Murine liver tissues were fixed in 4% paraformaldehyde, embedded in OCT, and sectioned into 8 μm frozen slices. Slices were air-dried, rinsed with PBS, and antigen-retrieved in citrate buffer (pH 6.0, 95 °C, 15 min). After blocking with 5% BSA (containing 0.3% Triton X-100, 1 h), slices were incubated overnight at 4 °C with primary antibodies (rabbit anti-GADD45G, 1:200; rat anti-F4/80, 1:150). After three washes with PBS-T, slices were incubated with Alexa Fluor 488-conjugated anti-rabbit IgG and Alexa Fluor 594-conjugated anti-rat IgG (1:500, 1 h, dark). Nuclei were stained with DAPI (5 min), and slices were mounted with anti-fade medium. Images were captured via confocal microscope (400×). Co-localization was quantified by Pearson’s correlation coefficient (r) using Zeiss ZEN software (version 3.2; 3 fields/section, 3 replicates/group).

### 2.16. Serum Biochemical Analysis

Serum samples were collected from mice by centrifugation of whole blood at 3000 rpm for 10 min at 4 °C and stored at −80 °C until analysis. The activities of serum alanine aminotransferase (ALT) and aspartate aminotransferase (AST) were measured using commercial assay kits (Nanjing Jiancheng Bioengineering Institute, Nanjing, China; catalog nos. C009-2-1 for ALT and C010-2-1 for AST) according to the manufacturer’s instructions. Absorbance was measured using a microplate reader (Thermo Fisher Scientific, Waltham, MA, USA), and enzyme activities were calculated and expressed as units per liter (U/L).

### 2.17. Identification of Candidate Compounds

Candidate compounds associated with GADD45G were identified via the Drug Signatures Database (DSigDB) available on the Enrichr platform (http://dsigdb.tanlab.org/DSigDBv1.0/; accessed on 10 July 2025). The three-dimensional structure of GADD45G was obtained from the Protein Data Bank (PDB ID: 3FFM). Structures of camptothecin and myristicin were obtained from PubChem. Molecular docking was performed using AutoDock Vina (version 1.2.5). Binding affinity was evaluated based on binding energy (kcal/mol), with values < −5.0 kcal/mol indicating stable interactions [37]. Docking results were visualized using PyMOL (version 2.5.5).

### 2.18. Isolation and Culture of Mouse Bone Marrow-Derived Macrophages (BMDMs)

Mouse BMDMs were isolated from the femurs and tibias of 6- to 8-week-old C57BL/6 mice. As previously described [38], bone marrow cells were flushed out with RPMI-1640 medium (Gibco, Grand Island, NY, USA) and centrifuged at 1000 rpm for 5 min. After discarding the supernatant, cells were resuspended in RPMI-1640 medium supplemented with 1% penicillin–streptomycin, 10% fetal bovine serum (Gibco, Grand Island, NY, USA), and 20 ng/mL macrophage colony-stimulating factor (M-CSF, PeproTech, Rocky Hill, NJ, USA), then seeded in 6-well plates at a density of 2 × 10^6^ cells/well. Cells were cultured at 37 °C in a 5% CO_2_ incubator for 7 days, with medium changed every 2 days to induce differentiation into mature BMDMs. Macrophage differentiation was confirmed by microscopic observation of cell morphology. Following 7 days of M-CSF induction, the cells exhibited characteristic mature macrophage features, including enlarged, fully adherent cell bodies with typical cell spreading characteristics [39]. For in vitro treatments, mature BMDMs were pretreated for 2 h with either camptothecin or myristicin. The concentrations used, 1 µM for camptothecin (Targetmol, China) and 50 µM for myristicin (Targetmol, Shanghai, China), were selected based on previously published studies demonstrating their biological activity without significant cytotoxicity [40,41]. Subsequently, the cells were exposed to 0.1 mM palmitic acid (PA; Sigma-Aldrich, St. Louis, MO, USA) for 24 h to induce lipotoxicity. A control group was treated with an equivalent volume of vehicle for the same duration.

### 2.19. Western Blotting

Liver tissues and BMDM were lysed with RIPA lysis buffer (Beyotime, Shanghai, China) including protease inhibitor and centrifuged at 12,000 rpm for 15 min at 4 °C. Based on the protein concentration determined by the BCA kit (Beyotime, Shanghai, China), samples were loaded by SDS–polyacrylamide gel electrophoresis, then transferred onto polyvinylidene difluoride membranes (Merck Millipore, Darmstadt, Germany). After blocking in 5% skim milk at room temperature for 1 h, membranes were incubated overnight at 4 °C with the following primary antibodies: GADD45G (1:1000, Invitrogen, Carlsbad, CA, USA); SOCS1 (1:1000, Invitrogen, Carlsbad, CA, USA); and SOCS2 (1:1000, Invitrogen, Carlsbad, CA, USA); β-actin (1:10,000, Proteintech, Wuhan, China); and GAPDH (1:5000, Proteintech, Wuhan, China). Then; membranes were incubated with the corresponding secondary antibodies for 1 h at room temperature. The bands were visualized using electrochemiluminescence (NCM, Suzhou, China) and recorded by an Integrated Chemiluminescence Gel Imaging System (Roche, Basel, Switzerland). The intensities of the bands were analyzed using ImageJ software (version, 1.53).

### 2.20. Statistical Analysis

All data are presented as means ± standard error of the mean (SEM). Unpaired *t*-tests and one-way analysis of variance (ANOVA) were performed using GraphPad Prism software (version 9.5) to assess significant differences between groups, with *p* < 0.05 considered statistically significant.

## 3. Results

### 3.1. Identification of DEGs, Functional Enrichment Analysis, and Immune Alteration in MASLD

**Figure 1:**
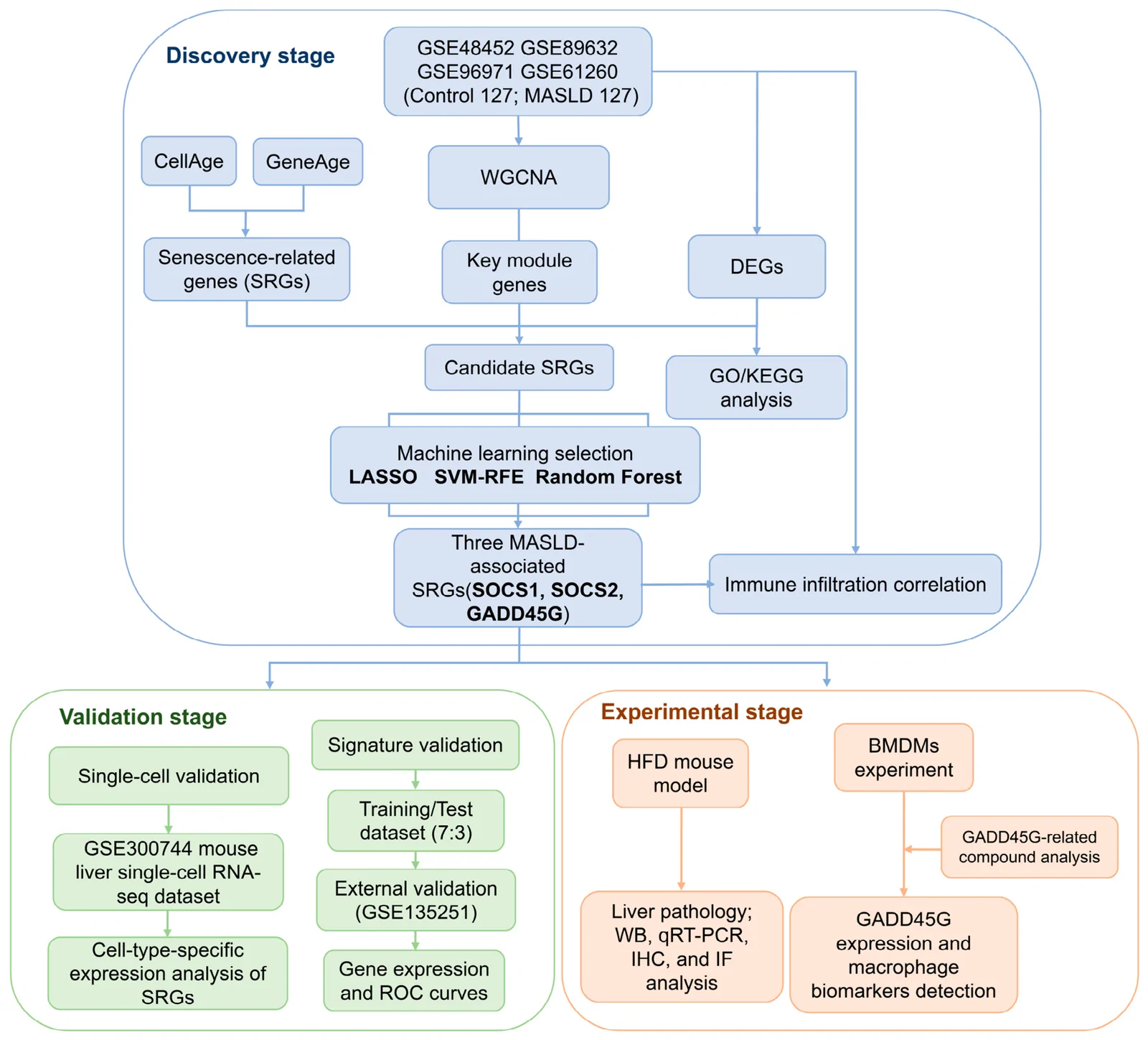
Overall study workflow. The study included three stages: discovery, validation, and experimental evaluation. In the discovery stage, four liver transcriptomic datasets (GSE48452, GSE89632, GSE96971, and GSE61260; 127 controls and 127 MASLD samples) were integrated to identify MASLD-associated SRGs using differential expression analysis, WGCNA, senescence-related gene databases (CellAge and GeneAge), and three machine learning algorithms (LASSO, SVM-RFE, and random forest). Immune infiltration analysis was performed to evaluate the association between core SRGs and immune alterations. In the validation stage, the SRG signature was assessed in internal training–testing cohorts, an external cohort (GSE135251), and single-cell RNA sequencing data (GSE300744). In the experimental stage, HFD-induced obese mice and PA-stimulated BMDMs were used to validate GADD45G expression patterns, macrophage-associated changes, and candidate compounds regulating GADD45G.

**Figure 2:**
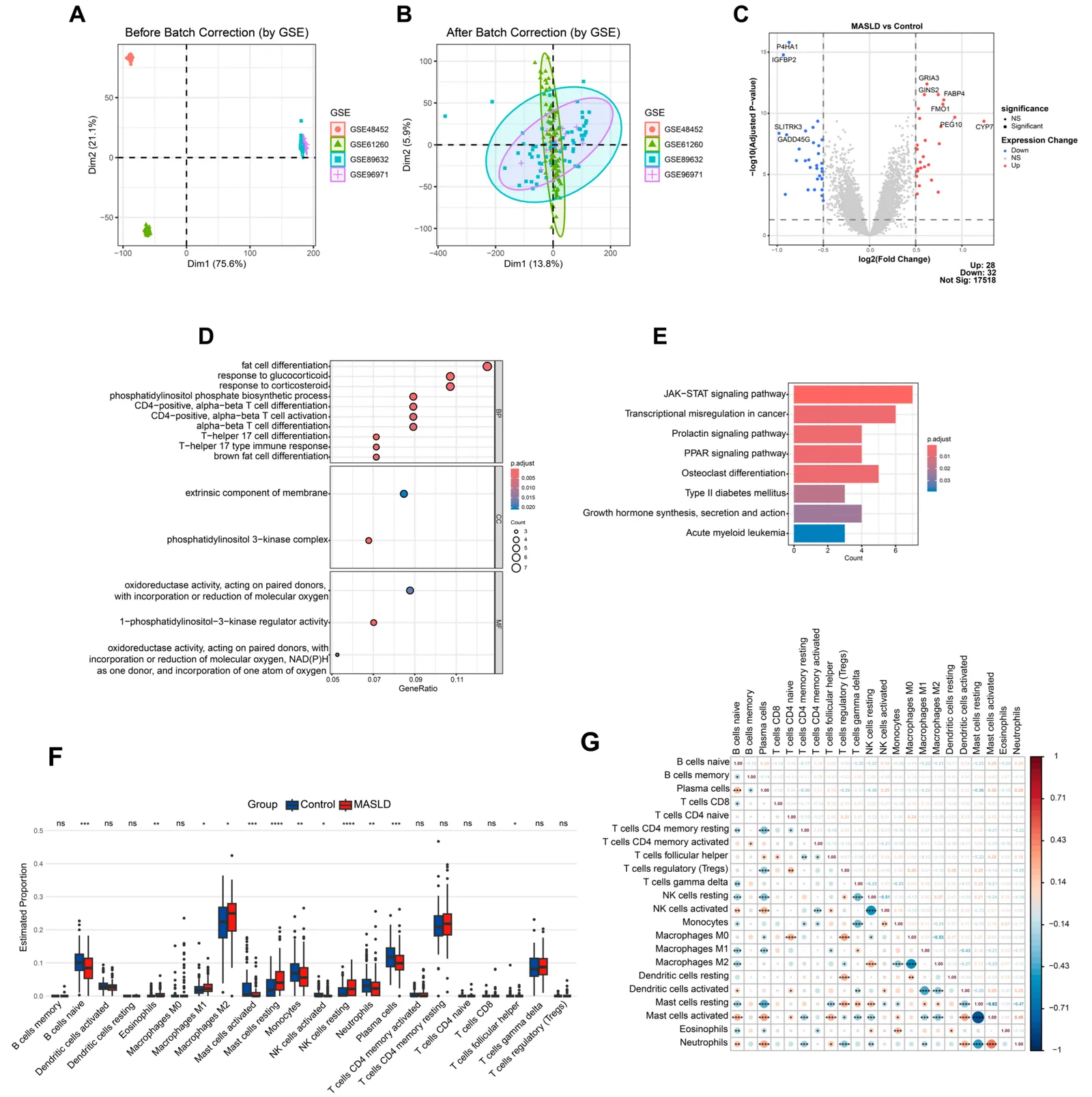
Integrated bioinformatic analysis of differentially expressed genes (DEGs) and immune landscape in MASLD. (**A**,**B**) Principal component analysis (PCA) plots before (**A**) and after (**B**) batch correction, confirming the elimination of technical variations across four merged GEO datasets. (**C**) Volcano plot illustrating 60 DEGs between MASLD and control samples, with 28 upregulated (red) and 32 downregulated (blue) genes identified. (**D**) Gene Ontology (GO) enrichment analysis of DEGs, covering biological processes, cellular components, and molecular functions. (**E**) Kyoto Encyclopedia of Genes and Genomes (KEGG) pathway enrichment analysis, highlighting key pathways involved in MASLD pathogenesis. (**F**) Box plots showing the proportion of 22 immune cell types between MASLD and control groups, analyzed via CIBERSORT. (**G**) Correlation heatmap of immune cell levels, revealing interrelationships among immune cell subsets in MASLD. ns: not significant, * *p* < 0.05, ** *p* < 0.01, *** *p* < 0.001, **** *p* < 0.0001.

The overall study workflow is shown in Figure 1. Batch effect correction was performed on the integrated GEO datasets, and PCA verified the successful removal of technical variations (Figure 2A,B). Boxplots and PCA plots illustrating data distribution before and after batch effect correction are presented in Supplementary Figure S1. Differential expression profiling identified a total of 60 differentially expressed genes in MASLD specimens, 28 upregulated and 32 downregulated genes, as displayed in the volcano plot (Figure 2C). GO enrichment analysis demonstrated that these DEGs were significantly enriched in biological processes closely associated with MASLD pathogenesis, such as fat cell differentiation and T cell activation (Figure 2D). KEGG pathway analysis further highlighted critical signaling cascades including JAK-STAT, PPAR, and type II diabetes mellitus pathways (Figure 2E). Immune infiltration analysis using CIBERSORT revealed elevated proportions of pro-inflammatory macrophages and activated dendritic cells, alongside reduced naïve B cells in MASLD (Figure 2F). A correlation heatmap further illustrated complex interactions among immune cell subsets, indicative of coordinated immune dysregulation in MASLD (Figure 2G).

Because GSE96971 lacked healthy control samples, a sensitivity analysis was performed by excluding this dataset and repeating the transcriptomic analysis. PCA showed comparable sample distribution after batch correction (Supplementary Figure S2A–D), and gene-level effect sizes between the original and sensitivity analyses showed strong concordance (Spearman correlation = 1.0) (Supplementary Figure S2E). Importantly, the expression trends of key candidate genes identified from the integrated analysis remained consistent after exclusion of GSE96971 (Supplementary Figure S2F and Table S2), supporting the robustness of the transcriptomic findings.

### 3.2. WGCNA for Identification of MASLD-Associated Key Modules

**Figure 3:**
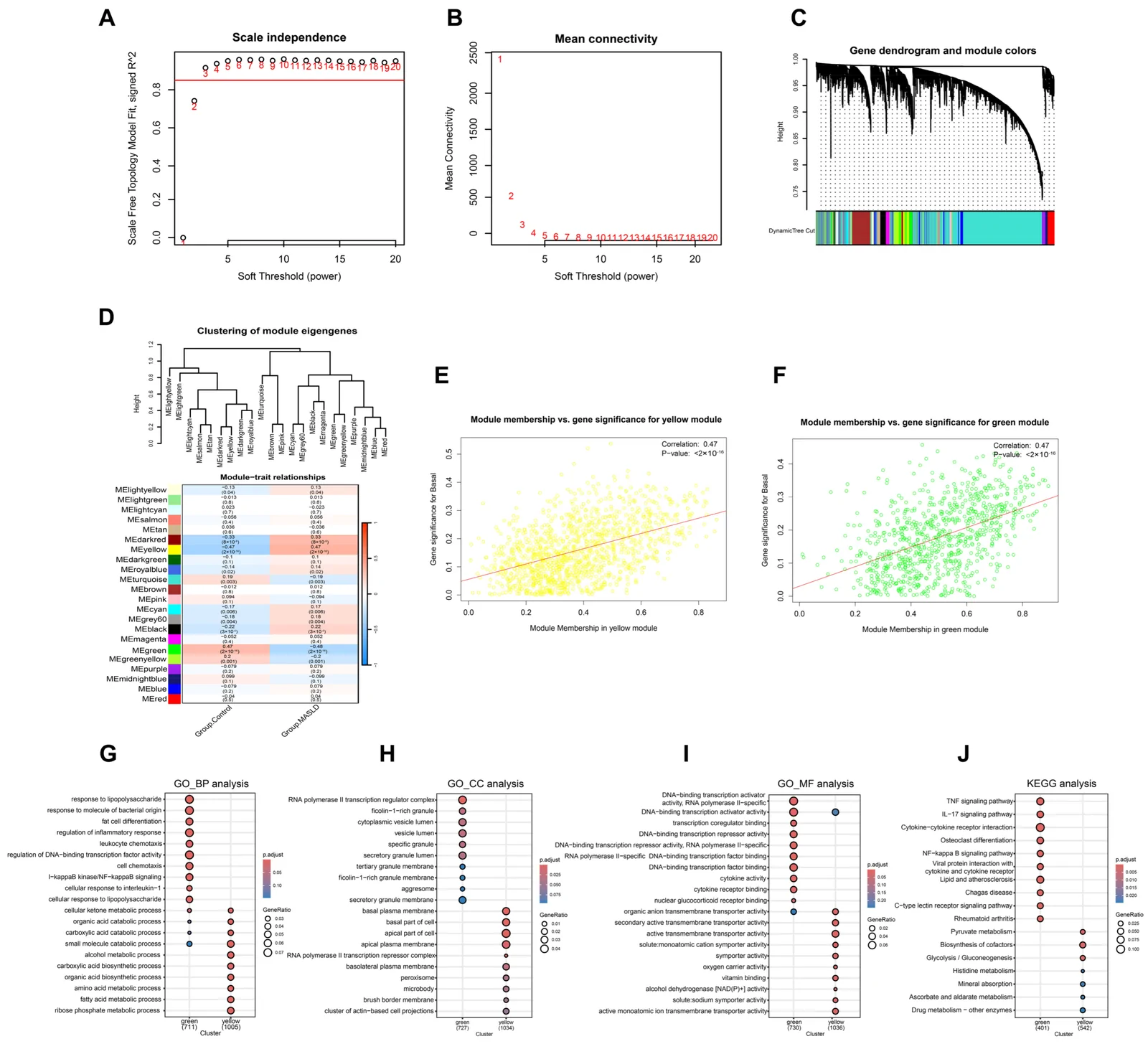
WGCNA identifies key gene modules associated with MASLD. (**A**,**B**) Selection of soft-threshold power for scale-free network construction. (**C**) Gene dendrogram and module color assignment. (**D**) Module–trait correlation heatmap, with the yellow module showing the strongest positive correlation and the green module showing the strongest negative correlation with MASLD. (**E**,**F**) Scatterplots of module membership vs. gene significance for the yellow and green modules. (**G**–**I**) GO enrichment analysis (BP, CC, MF) of hub genes in key modules. (**J**) KEGG pathway enrichment analysis of hub genes, highlighting key inflammatory and metabolic pathways in MASLD.

To identify gene networks linked to MASLD, we performed WGCNA, selecting a soft-threshold power of β = 3 to establish a scale-free network (Figure 3A,B). A total of 22 co-expression modules were constructed (Figure 3C). Module–trait correlation analysis revealed the yellow module had the strongest positive correlation and the green module the strongest negative correlation with MASLD status (Figure 3D). Scatterplots confirmed significant positive correlations between module membership and gene significance for both modules (r = 0.47, *p* < 0.001), indicating their relevance (Figure 3E,F).

Functional enrichment of hub genes in key modules showed significant enrichment in immune activation, inflammatory response, and lipid metabolism-related GO terms (Figure 3G–I). KEGG analysis further highlighted critical pathways, including TNF, NF-κB, and cytokine–cytokine receptor interaction, potentially involved in MASLD pathogenesis (Figure 3J). Genes from these two modules and DEGs were selected for subsequent intersection with SRGs.

### 3.3. Screening of Core SRGs via Machine Learning and Construction of Three-Gene MASLD-Associated Molecular Signature

**Figure 4:**
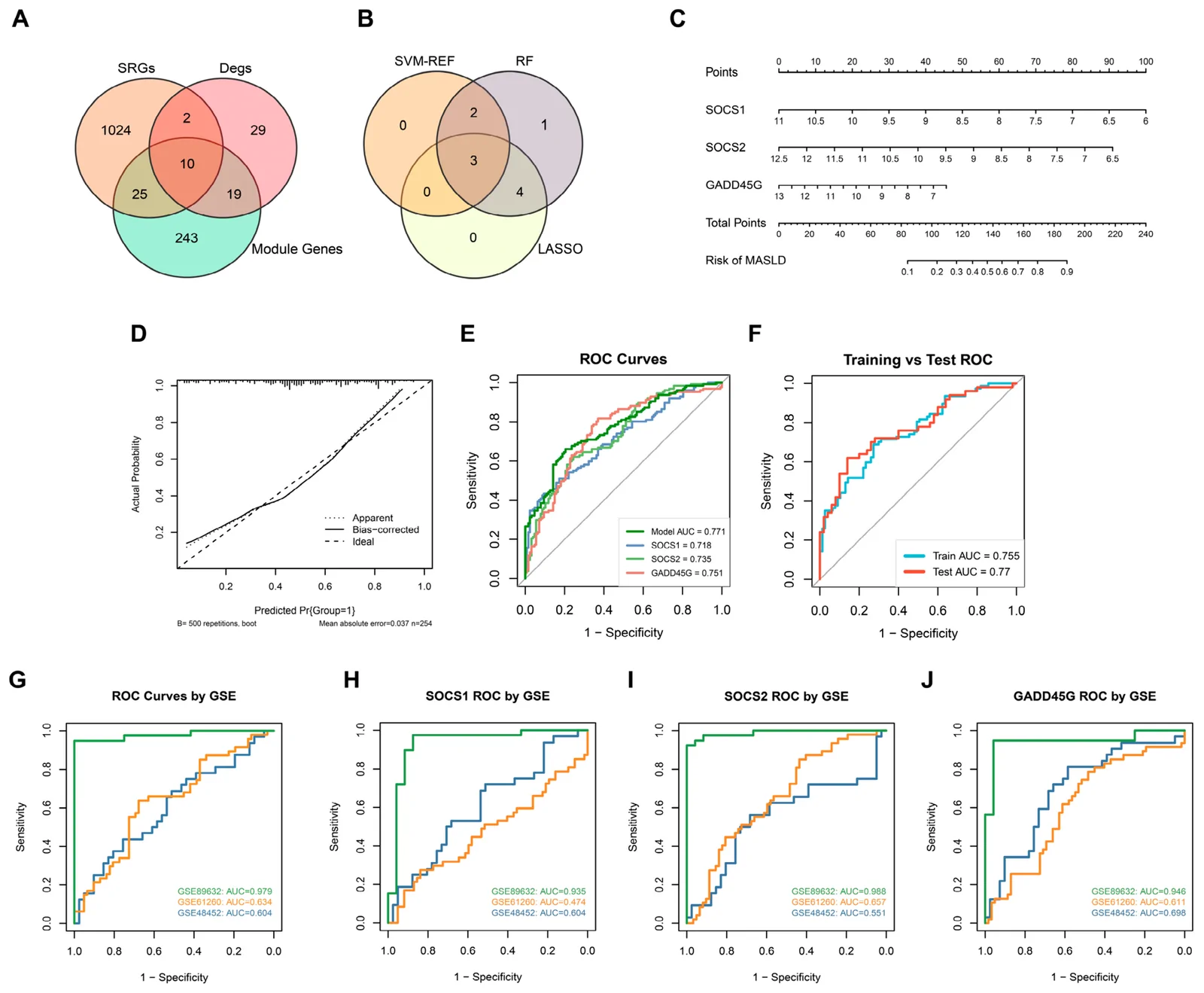
Identification and evaluation of the senescence-related gene (SRG) signature associated with MASLD. (**A**) Venn diagram showing the intersection of SRGs, DEGs, and WGCNA hub genes. (**B**) Venn diagram of hub genes screened by three machine learning algorithms. (**C**) Nomogram constructed based on SOCS1, SOCS2, and GADD45G expression levels to estimate the risk of MASLD. (**D**) Calibration curve of three-gene MASLD-associated molecular signature. (**E**) ROC curves of the three-gene signature and individual genes in the internal datasets. (**F**) ROC curves of the three-gene signature after splitting the internal datasets into a training set and a testing set in a 7:3 ratio. (**G**) ROC curves of the three-gene signature in 3 independent GEO datasets (GSE89632, GSE61260, and GSE48452). (**H**–**J**) ROC curves of core candidate SRGs, namely SOCS1 (**H**), SOCS2 (**I**), and GADD45G (**J**), in 3 independent GEO datasets (GSE89632, GSE61260, and GSE48452).

To identify candidate SRGs associated with MASLD, we intersected SRGs, DEGs, and module genes, yielding 10 overlapping candidate genes (Figure 4A). Three machine learning algorithms (SVM-RFE, RF, LASSO) further screened three key SRGs: SOCS1, SOCS2, and GADD45G (Figure 4B). A three-gene molecular signature was constructed, and the calculated risk scores showed a gradual increase in MASLD-associated scores across samples (Figure 4C). Calibration curves confirmed good consistency between predicted and actual MASLD probabilities (Figure 4D). ROC analysis showed the three-gene signature achieved an AUC of 0.771, with individual AUCs of 0.718 for SOCS1, 0.735 for SOCS2, and 0.751 for GADD45G (Figure 4E). The signature showed consistent performance in both training (AUC = 0.755) and testing (AUC = 0.77) cohorts, split 7:3 from the integrated dataset (Figure 4F). Furthermore, independent GEO cohorts (GSE89632, GSE61260, and GSE48452) were used to further evaluate the robustness of the three-gene signature and individual SRGs (Figure 4G–J).

### 3.4. Association of Core SRGs with Clinical Features and Immune Characteristics

**Figure 5:**
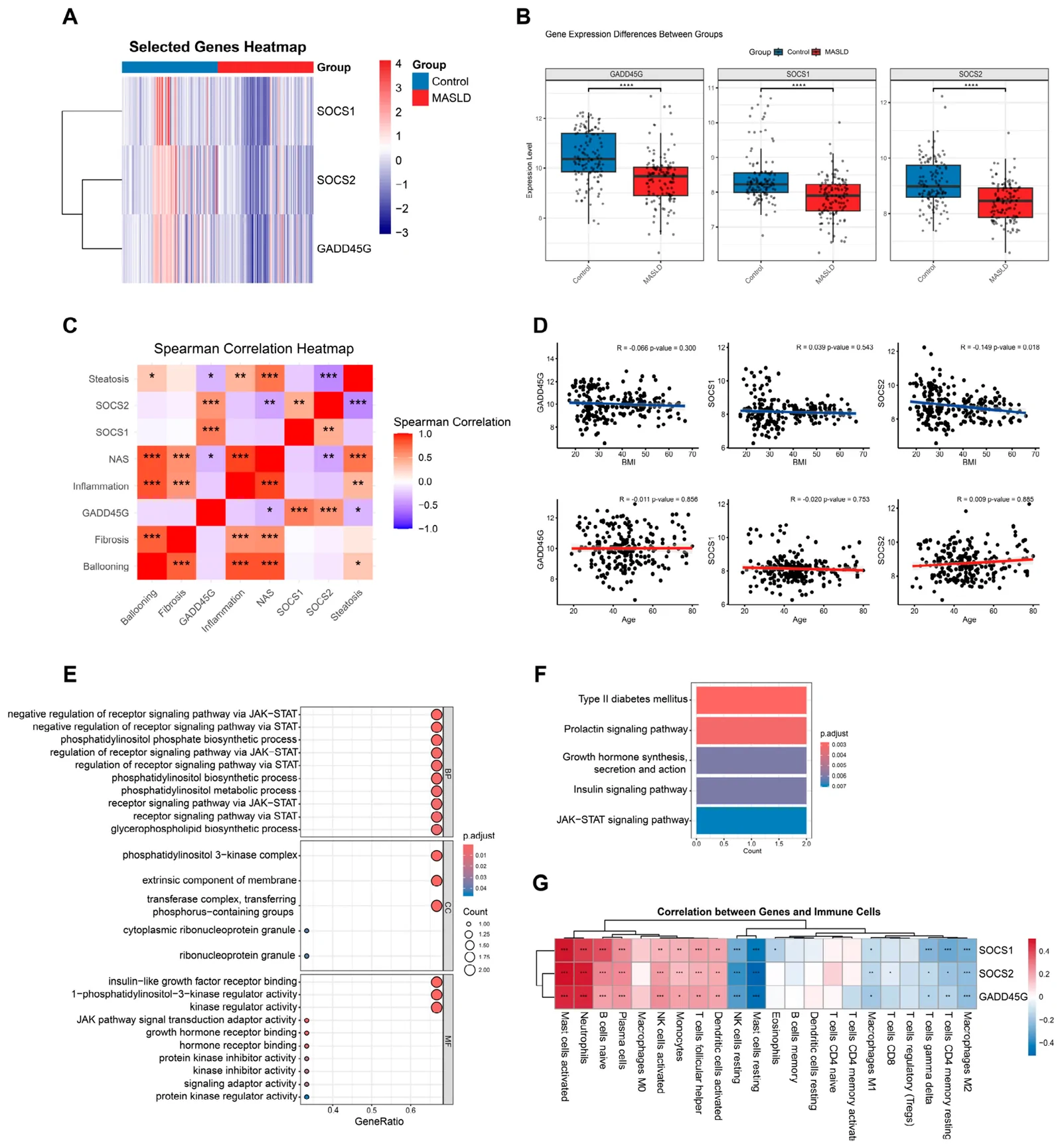
Expression patterns, clinical correlation, functional analysis and immune correlations of the candidate SRGs in MASLD. (**A**) Heatmap showing the expression profiles of the three SRGs in control and MASLD samples. (**B**) Box plots confirming significantly downregulated expression of SOCS1, SOCS2, and GADD45G in MASLD. (**C**) Spearman correlation heatmap illustrating associations between the genes and MASLD histological features (steatosis, inflammation, NAS, fibrosis, ballooning). (**D**) Scatterplots showing no significant correlations between the expression of the three core genes and BMI or age. (**E**,**F**) GO and KEGG functional enrichment analysis of the key genes, highlighting JAK-STAT, insulin signaling, and type II diabetes mellitus pathways. (**G**) Correlation heatmap demonstrating associations between the three genes and immune cell populations estimated by CIBERSORT. Statistical significance was determined using Student’s *t*-test. * *p* < 0.05, ** *p* < 0.01, *** *p* < 0.001, **** *p* < 0.0001.

The heatmap showed downregulation of SOCS1, SOCS2, and GADD45G in MASLD samples relative to controls (Figure 5A). Box plots further verified significantly reduced expression of all three genes in MASLD (Figure 5B). Spearman correlation analysis revealed associations between these genes and MASLD histological features, including steatosis, inflammation, NAS score, and fibrosis (Figure 5C). Scatterplots demonstrated no significant correlations between the expression of the three core genes and BMI or age (Figure 5D). GO enrichment analysis showed these genes were primarily involved in JAK-STAT signaling regulation, receptor signaling pathways, and metabolic processes, while KEGG analysis highlighted key pathways, including JAK-STAT, insulin signaling, and type II diabetes mellitus, suggesting their association with metabolic and immune-related processes in MASLD (Figure 5E,F). Immune correlation analysis further showed significant associations between the three genes and multiple immune cell populations, including macrophage-related subsets (Figure 5G).

### 3.5. Core SRG Expression in External GEO Dataset and HFD-Induced MASLD Mice

**Figure 6:**
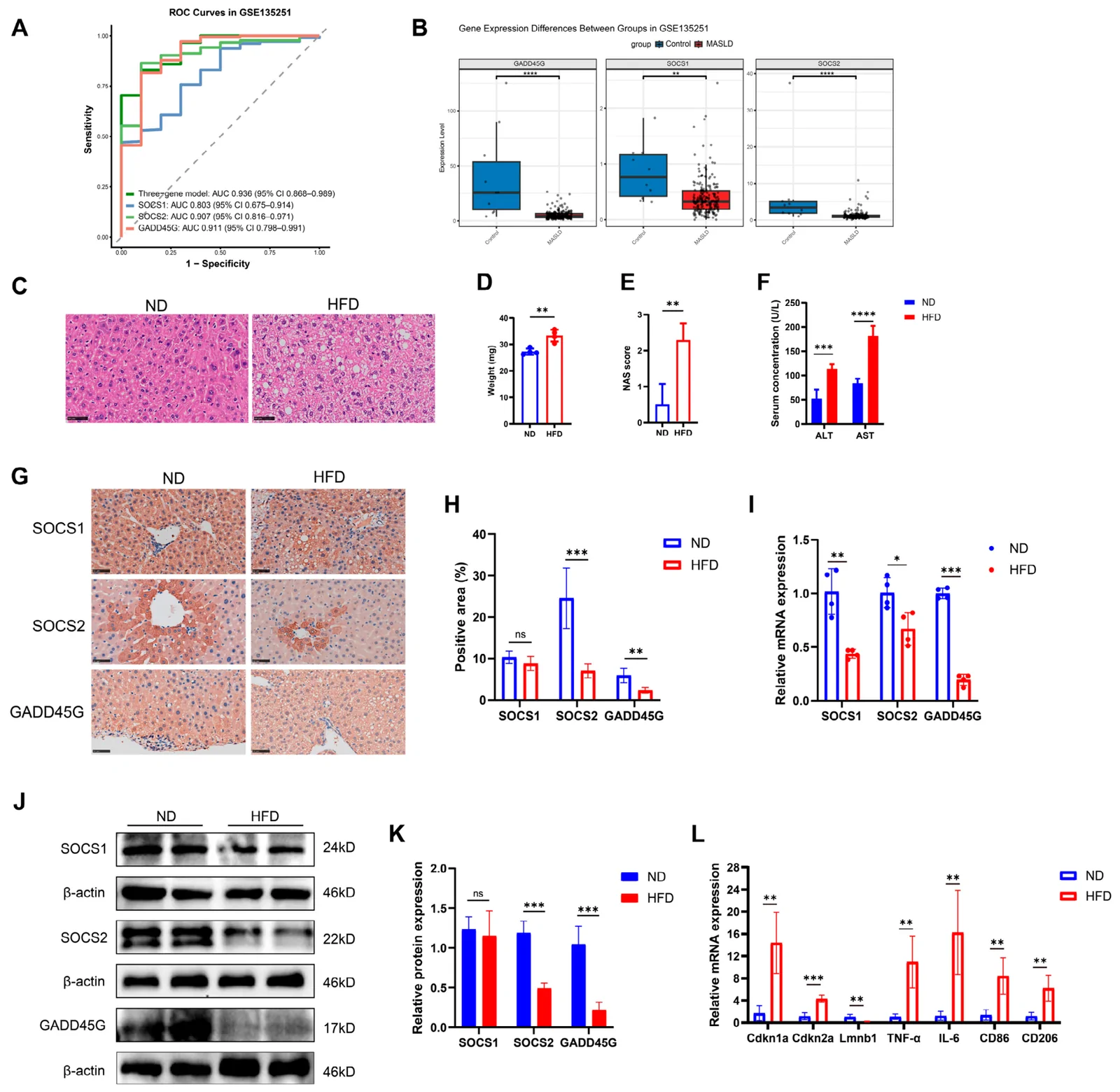
External validation of the SRG signature and experimental validation of the three-gene expression in MASLD models. (**A**) ROC curves of the three-gene signature and individual genes (SOCS1, SOCS2, GADD45G) in the independent GSE135251 cohort. (**B**) Box plots showing significantly downregulated expression of the three SRGs in MASLD samples from GSE135251. (**C**) H&E staining of liver tissues from normal diet (ND) and high-fat diet (HFD) mice (scale bar: 50 μm). (**D**) Body weight comparison between ND- and HFD-fed mice. (**E**) NAS scores of liver tissues from ND- and HFD-fed mice. (**F**) Serum ALT and AST levels in ND- and HFD-fed mice. (**G**) Immunohistochemical staining of SOCS1, SOCS2, and GADD45G in mouse liver tissues (scale bar: 50 μm). (**H**) Quantitative analysis of immunohistochemically positive area. (**I**) Relative mRNA expression of SOCS1, SOCS2, and GADD45G in ND and HFD mice (**J**) Western blot analysis of protein expression levels in ND and HFD mice. (**K**) Quantitative analysis of Western blot protein expression. (**L**) Relative expression of senescence-associated and macrophage inflammatory markers, including Cdkn1a, Cdkn2a, Lmnb1, TNF-α, IL-6, CD86, and CD206, in liver tissues. Data are presented as means ± SEM. Statistical significance was determined using Student’s *t*-test. * *p* < 0.05, ** *p* < 0.01, *** *p* < 0.001, **** *p* < 0.0001.

External validation using the independent GSE135251 dataset further supported the classification performance of the three SRGs: SOCS1, SOCS2, and GADD45G. The three-gene signature achieved an AUC of 0.936 (95% CI: 0.868–0.989), while the individual AUCs were 0.803 for SOCS1 (95% CI: 0.675–0.914), 0.907 for SOCS2 (95% CI: 0.816–0.971), and 0.911 for GADD45G (95% CI: 0.798–0.991) (Figure 6A and Supplementary Table S3). Precision–recall curve analysis further supported the robustness of the three-gene signature in GSE135251 dataset (Supplementary Figure S3A). Moreover, the three-gene signature achieved a sensitivity of 83.01% and a specificity of 90.00% in the GSE135251 cohort (Supplementary Table S3). Box plot analysis showed that all three SRGs were significantly downregulated in MASLD samples compared with controls (Figure 6B).

To experimentally validate the bioinformatic findings, an HFD-induced metabolic dysfunction model was established in mice. Compared with ND-fed mice, HFD-fed mice exhibited increased body weight, hepatic steatosis, elevated NAS scores, and increased serum ALT and AST levels, confirming successful establishment of the metabolic liver injury model (Figure 6C–F).

Immunohistochemistry revealed altered expression patterns of core candidate SRGs in HFD-fed mice. Among the three genes, GADD45G showed a marked reduction in hepatic expression accompanied by decreased SOCS2 expression, whereas SOCS1 protein staining showed no significant difference among groups (Figure 6G,H). qRT-PCR analysis demonstrated reduced hepatic mRNA levels of SOCS1, SOCS2, and GADD45G following HFD feeding (Figure 6I). Western blot analysis further confirmed decreased protein expression of SOCS2 and GADD45G in HFD liver tissues, while SOCS1 protein levels remained unchanged (Figure 6J,K).

Because MASLD progression is closely associated with inflammatory activation and cellular stress responses, we further examined senescence-associated and macrophage-related inflammatory markers. HFD feeding increased the expression of Cdkn1a, Cdkn2a, TNF-α, IL-6, CD86, and CD206, indicating enhanced inflammatory and stress-associated responses in the liver (Figure 6L). Together, these findings support the association of the SRG signature with MASLD-related molecular alterations and inflammatory responses.

### 3.6. Identification of GADD45G-Associated Compounds and BMDM Validation

To further characterize the cellular distribution of GADD45G in the liver, single-cell RNA sequencing analysis was performed using the GSE300744 dataset. UMAP clustering identified major hepatic cell populations, and macrophage populations were annotated based on canonical markers, including Adgre1, Lyz2, C1qa, Clec4f, Ccr2, and Ly6c2 (Supplementary Figure S4A,B). GADD45G expression was detected across different liver cell populations and showed macrophage-associated expression patterns (Supplementary Figure S4C,D).

**Figure 7:**
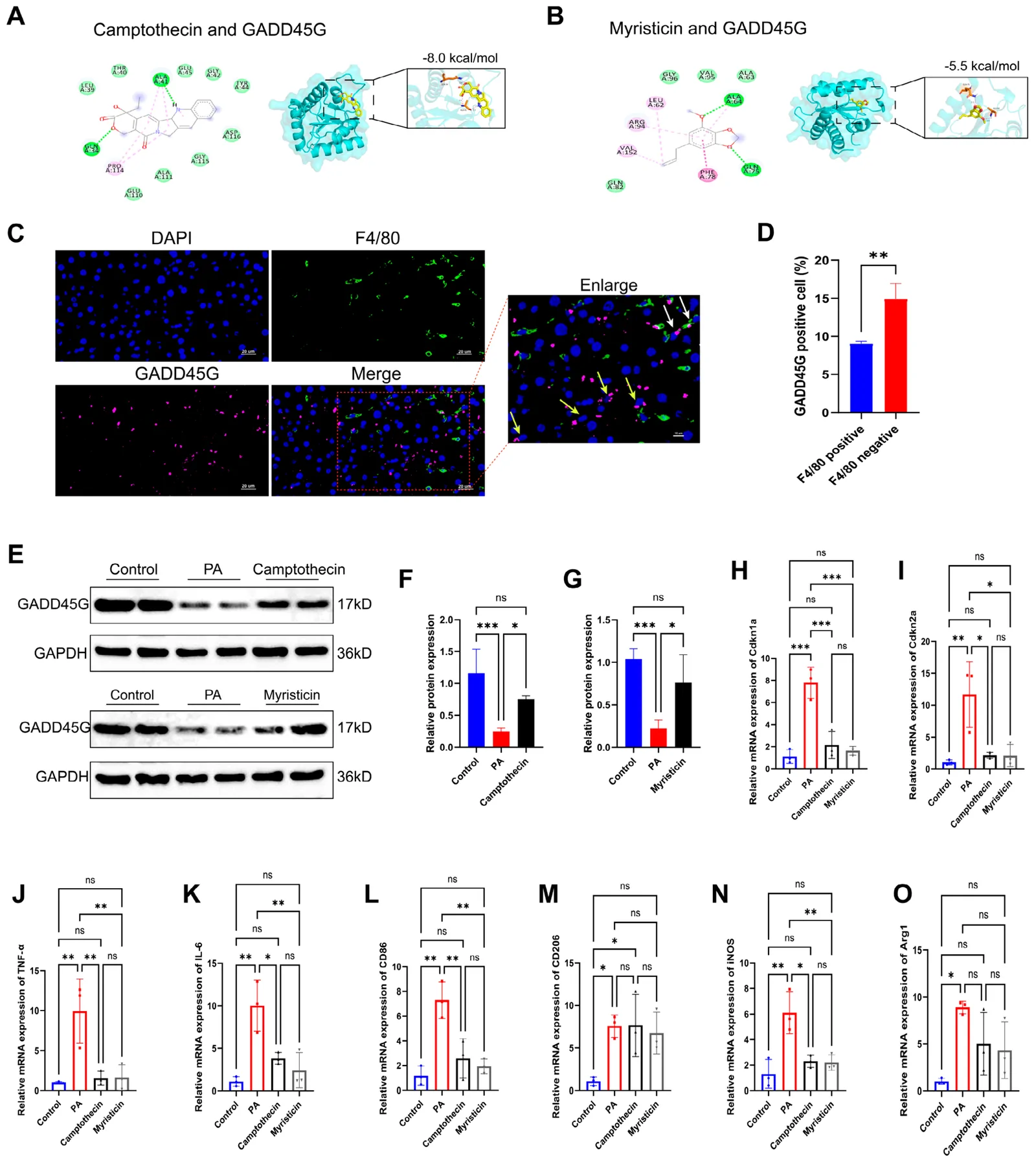
Molecular docking and functional validation of GADD45G in BMDMs. (**A**,**B**) Molecular docking analysis showing the predicted interactions between GADD45G and candidate compounds camptothecin and myristicin. (**C**) Representative immunofluorescence images showing GADD45G and F4/80 expression in ND-fed mouse liver tissues. Yellow arrows indicate F4/80-negative possible GADD45G localization, whereas white arrows indicate F4/80-positive GADD45G localization. (**D**) Quantification of GADD45G-positive cells among F4/80-positive and F4/80-negative populations. (**E**–**G**) Western blot analysis and quantification of GADD45G protein expression in PA-stimulated BMDMs treated with camptothecin or myristicin. (**H**–**O**) Expression analysis of senescence-associated and macrophage inflammatory markers following PA stimulation and compound treatment. Data are presented as means ± SEM. Statistical significance was determined using Student’s *t*-test. * *p* < 0.05, ** *p* < 0.01, *** *p* < 0.001.

To further investigate the regulation of GADD45G under metabolic stress, molecular docking analysis identified potential interactions between GADD45G and two candidate compounds, camptothecin and myristicin (Figure 7A,B), with binding energies of −8.0 and −5.5 kcal/mol, respectively. Immunofluorescence analysis revealed that GADD45G was detected in both F4/80-positive macrophages and F4/80-negative cells. Quantitative analysis showed a higher proportion of GADD45G-positive cells in the F4/80-negative population than in F4/80-positive macrophages (Figure 7C,D).

In PA-stimulated BMDMs, GADD45G protein expression was markedly reduced, whereas treatment with camptothecin or myristicin partially restored GADD45G expression (Figure 7E–G). Moreover, these compounds partially reversed PA-induced changes in senescence-associated markers (Cdkn1a and Cdkn2a) and inflammatory markers associated with macrophage activation (TNF-α, IL-6, and iNOS) (Figure 7H–O). Together, these findings support that GADD45G is associated with macrophage-related immune alterations in MASLD and can be modulated under metabolic stress conditions. However, whether the effects of these compounds are directly mediated through GADD45G requires further genetic validation.

## 4. Discussion

**Figure 8:**
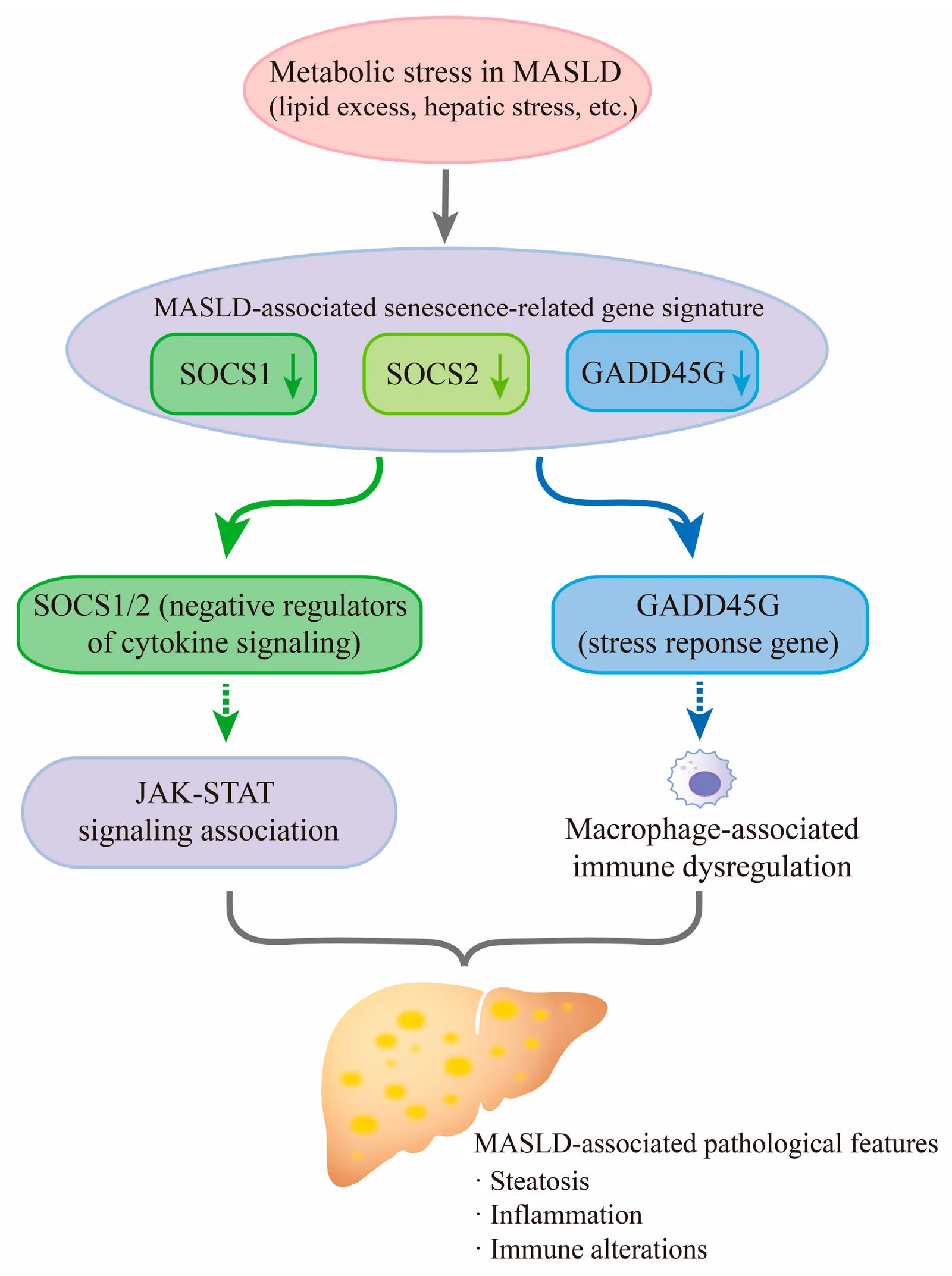
Proposed working model summarizing the association between SRGs and immune dysregulation in MASLD. Metabolic stress associated with MASLD, including lipid overload and hepatic stress, is associated with reduced expression of SRGs (SOCS1, SOCS2, and GADD45G). SOCS1 and SOCS2 are associated with cytokine signaling-related pathways, whereas GADD45G shows macrophage-associated expression patterns and potential involvement in immune alterations. Green and blue arrows indicate SOCS1/2- and GADD45G-related pathways, respectively, whereas gray arrows represent the downstream convergence toward MASLD-associated pathological features. Dashed arrows indicate potential associations. Together, these senescence-related molecular changes are associated with MASLD-related pathological features, including steatosis, inflammation, and immune alterations.

In the present study, we employed an integrated strategy encompassing transcriptomic integration, machine learning algorithms, external dataset validation, single-cell analysis, and in vivo/in vitro experimental models to identify and evaluate three SRGs, namely SOCS1, SOCS2, and GADD45G, as candidate senescence-related molecular signatures associated with MASLD biology. The expression of these genes was downregulated in MASLD liver tissues, correlating with clinical severity, histological steatosis, and immune-related alterations. Furthermore, single-cell RNA sequencing and immunofluorescence staining revealed macrophage-associated localization of GADD45G in MASLD liver. In PA-stimulated BMDMs, downregulation of GADD45G accompanied heightened inflammatory and senescence-associated responses, whereas exploratory chemical probes (camptothecin and myristicin) partially restored GADD45G expression and attenuated these metabolic stress responses. Together, these findings provide preliminary evidence linking senescence-related gene dysregulation to hepatic immune stress in MASLD. A proposed working model summarizing the potential relationship between MASLD-associated metabolic stress, SRG dysregulation, and immune alterations is presented in Figure 8.

SOCS1 and SOCS2 are well established as indispensable negative-feedback regulators of cytokine signaling, primarily through the inhibition of the Janus kinase–signal transducer and activator of transcription (JAK-STAT) pathway [42]. The observed downregulation of these genes in MASLD may contribute to the altered inflammatory regulation that characterizes the disease. In a healthy state, SOCS proteins act as negative regulators on cytokine signaling, helping to prevent excessive inflammatory responses [42,43]. Their diminished expression, as seen in our data, likely dismantles this crucial homeostatic checkpoint, leading to unchecked STAT activation and the perpetuation of pro-inflammatory cascades. This interpretation is supported by enrichment analyses that revealed associations with immune response and signaling pathways. This finding aligns with the broader understanding that failure in inflammation resolution is a key driver of liver injury in MASLD [4].

Our study identified GADD45G as another pivotal downregulated gene. As a member of the growth arrest and DNA damage-inducible (GADD45) family, it is a canonical sensor of cellular stress, orchestrating responses to genotoxic, oxidative, and metabolic insults to maintain cellular homeostasis [44]. Within the lipotoxic environment of the MASLD liver, hepatocytes are under constant metabolic stress, which can lead to DNA damage and organelle dysfunction [8]. Reduced expression of GADD45G may therefore critically impair the cell’s adaptive capacity to repair damage and manage stress, thereby lowering the threshold for cellular injury and apoptosis [45]. Furthermore, reduced expression of GADD45G in hepatic macrophages may impair adaptive stress responses, thereby exacerbating inflammatory signaling and senescence-associated alterations. Our findings in PA-treated BMDMs demonstrate that changes in GADD45G expression are associated with altered macrophage inflammatory responses and senescence-associated marker expression. Whether GADD45G directly regulates macrophage polarization requires further genetic rescue studies, these results highlight GADD45G as a potential key link between metabolic stress and macrophage dysregulation in MASLD.

The interplay between cellular senescence, stress responses, and inflammation likely forms a self-perpetuating pathogenic loop in MASLD [4]. Chronic metabolic stress can induce a state of cellular senescence in hepatocytes and other liver cells, characterized by the secretion of SASP [46]. The SASP components (e.g., IL-6, TNF-α) recruit immune cells and fuel local inflammation [13,46]. Our findings suggest that altered expression of SRGs may be associated with this stress–inflammation network. Reduced GADD45G expression may reflect impaired cellular stress adaptation under metabolic stress conditions, potentially increasing susceptibility to senescence-associated alterations. Downregulation of SOCS1/SOCS2 may affect inflammatory signaling regulation, which could further contribute to the inflammatory environment in MASLD. Together, these findings suggest that dysregulation of SRGs may be involved in the progression of MASLD, although the underlying causal mechanisms require further investigation.

From a translational perspective, our identification of camptothecin and myristicin as compounds associated with GADD45G expression provides valuable tools for future research. Camptothecin, a cytotoxic DNA damage agent used in cancer therapy [47], was identified as a compound associated with GADD45G expression changes in macrophages under metabolic stress conditions. Myristicin, a natural product from nutmeg with a complex toxicological and anti-inflammatory profile [40,41], was also found to regulate GADD45G. Although the precise mechanisms and functional consequences of GADD45G modulation by these compounds were not determined in this study, these findings suggest that camptothecin and myristicin may serve as exploratory chemical probes to further investigate the role of GADD45G in macrophage-associated responses under metabolic stress conditions. Further genetic and pharmacological studies are required to determine whether these effects are directly mediated through GADD45G and whether they have therapeutic relevance in MASLD.

Notably, while SOCS1, SOCS2, and GADD45G were all downregulated at the mRNA level, only SOCS2 and GADD45G protein levels were significantly reduced. This is likely attributable to complex post-transcriptional and post-translational regulatory mechanisms. SOCS1, for example, is notoriously subject to rapid, ubiquitin-mediated proteasomal degradation, which can uncouple its mRNA and protein expression levels [42,48]. This highlights the importance of multi-omic analysis to capture the full regulatory landscape of these signaling molecules.

Several limitations of this study should be acknowledged. First, our transcriptomic analyses were based on publicly available GEO datasets and may be affected by inter-study heterogeneity and platform-related variation. Although batch correction and additional validation analyses supported the robustness of our findings, differences in cohort composition, including the imbalanced case–control ratio and disease spectrum in the external GSE135251 cohort, should be considered. Further validation in larger and more balanced cohorts is warranted. Second, immune cell proportions inferred by CIBERSORT from bulk liver transcriptomes should be interpreted cautiously, as changes in hepatic cellular composition during MASLD progression may influence immune deconvolution results. Therefore, these findings may reflect overall tissue remodeling rather than direct immune infiltration. Single-cell RNA sequencing analysis (GSE300744) was further incorporated to support macrophage-associated expression patterns of GADD45G. Third, although Cdkn1a and Cdkn2a were evaluated as senescence-associated markers in our models, comprehensive assessment of cellular senescence, including SA-β-gal activity, SASP profiling, DNA damage markers, and senescent cell burden, was not performed. Therefore, our findings should be interpreted as reflecting senescence-associated responses rather than definitive establishment of a senescent phenotype. In addition, the functional roles of SOCS1 and SOCS2, as well as the mechanistic contribution of GADD45G to macrophage-associated responses, require further investigation using genetic gain- and loss-of-function approaches. Fourth, camptothecin and myristicin were evaluated as exploratory chemical probes rather than therapeutic agents. Their mechanisms of action, relationship with GADD45G regulation, and safety profiles require further validation in comprehensive experimental models. Finally, although the 16-week HFD model effectively recapitulates metabolic stress and hepatic steatosis, it does not fully reproduce advanced MASH features, such as extensive inflammation and fibrosis.

## 5. Conclusions

In conclusion, SOCS1, SOCS2, and GADD45G represent a candidate senescence-related molecular signature associated with MASLD and immune-related alterations. The macrophage-associated reduction of GADD45G and its modulation under metabolic stress conditions provide preliminary evidence linking senescence-associated responses with macrophage-related changes in MASLD. However, further studies using comprehensive senescence assessments and functional genetic approaches are required to determine the causal roles of these genes in MASLD-associated senescence biology.

## Figures and Tables

**Table 1:** Dataset details utilized in the study.

Dataset	Platform	Sample Size		Organism	Tissue	Attribute
		Healthy Control	MASLD			
GSE48452	GPL11532	41	32	Human	Liver	Training/Test
GSE89632	GPL14951	24	39	Human	Liver	Training/Test
GSE61260	GPL11532	62	47	Human	Liver	Training/Test
GSE96971	GPL14951	0	9	Human	Liver	Training/Test
GSE135251	GPL18573	10	206	Human	Liver	Validation

**Table 2:** The primer sequences utilized in the study.

Gene	Forward Primer	Reverse Primer
*β-actin*	ACTGTCGAGTCGCGTCC	CTGACCCATTCCCACCATCA
*GADD45G*	CTGCAGATCCATTTCACGTTGAT	AGGATTCGAAATGAGGATGCAATG
*SOCS1*	CAACGGAACTGCTTCTTCGC	AGCTCGAAAAGGCAGTCGAA
*SOCS2*	CGCGAGCTCAGTCAAACAG	ACTCAATCCGCAGGTTAGTCG
*Cdkn1a*	TGGAGACCTGATGATACCCAACTA	GAAGAGACAACGGCACACTTTG
*Cdkn2a*	CTCTGGCTTTCGTGAACATGTTG	GCACCGTAGTTGAGCAGAAGAG
*Lmnb1*	GAGGAAAGCGGAAGAGAGTTGAT	GTTGATCCTGCTCAGAAGTGTTC
*TNF-α*	CCCTCACACTCACAAACCAC	ATAGCAAATCGGCTGACGGT
*IL-6*	CTTCTTGGGACTGATGCTGGTGAC	AGGTCTGTTGGGAGTGGTATCCTC
*CD86*	ACGGAGTCAATGAAGATTTCCT	GATTCGGCTTCTTGTGACATAC
*CD206*	CTCTGTTCAGCTATTGGACGC	CGGAATTTCTGGGATTCAGCTTC
*iNOS*	GAGACAGGGAAGTCTGAAGCAC	CCAGCAGTAGTTGCTCCTCTTC
*Arg1*	GTACATTGGCTTGCGAGACG	CGGCCTTTTCTTCCTTCCCAG

## Data Availability

All animal and cell experimental data generated in this study are included within this article. The GSE48452, GSE89632, GSE96971, GSE61260, GSE135251, and GSE300744 datasets are accessible from the public GEO database.

## Supplementary Materials

The following supporting information can be downloaded at https://www.mdpi.com/article/10.3390/biom16081154/s1, Figure S1: Assessment of batch effect correction in integrated GEO datasets; Figure S2: Sensitivity analysis demonstrates the robustness of transcriptomic integration after exclusion of the MASLD-only dataset; Figure S3: Precision–recall curve analysis of the SRG signature in the GSE135251 cohort; Figure S4: Single-cell RNA sequencing analysis of Gadd45g expression patterns in mouse liver; Table S1: Senescence-related genes obtained from CellAge and GenAge databases; Table S2: Differential expression consistency of core SRGs before and after exclusion of the GSE96971 dataset; Table S3: Classification performance of the three-gene SRG signature in the independent GSE135251 validation cohort. Supplementary File S1: Original Western blot images.
