# Supplementary material for: Senescence-Related SOCS1, SOCS2, and GADD45G Identify an Immune-Associated Molecular Signature in Metabolic Dysfunction-Associated Steatotic Liver Disease
Source: Biomolecules. 2026 Aug 7;16(8):1154. doi: 10.3390/biom16081154 (PMC13510971; doi:10.3390/biom16081154)
Supplement: Supplementary file 1 [file biomolecules-16-01154-s001.zip › biomolecules-4383383 - supplementary v6/biomolecules-4383383-supplementary.pdf]

Supplementary Table S1. Senescence-related genes identified from CellAge and GenAge databases.

No.	Gene.symbol	No.	Gene.symbol	No.	Gene.symbol	No.	Gene.symbol
1	AAK1	266	FOXP1	531	NRF1	796	TP53BP1
2	ABCB1	267	FOXP3	532	NRSN2	797	TP53BP2
3	ABCC6	268	FOXQ1	533	NSUN2	798	TP53I3
4	ABI3	269	FSCN1	534	NTN4	799	TP53INP1
5	ABI3BP	270	FXN	535	NUAK1	800	TP63
6	ACER2	271	FXR1	536	NUAK2	801	TPP1
7	ACKR1	272	G6PD	537	NUDT5	802	TPR
8	ACLY	273	GADD45G	538	NUTF2	803	TPX2
9	ADCK5	274	GAPDH	539	OGG1	804	TRA2B
10	AGO2	275	GATA4	540	OGT	805	TRDMT1
11	AGR2	276	GDF15	541	OPA1	806	TRIM28
12	AGT	277	GEMIN2	542	ORAI1	807	TRPM7
13	AHR	278	GGCT	543	ORC1	808	TRPM8
14	AKAP4	279	GJA1	544	OTX2	809	TRRAP
15	AKR1B1	280	GKN1	545	OXTR	810	TSC22D1
16	AKT1	281	GLI1	546	P2RY14	811	TTK
17	AKT1S1	282	GMNN	547	P3H1	812	TWIST1
18	AKT3	283	GMPS	548	PAK2	813	TWIST2
19	AKTIP	284	GNG11	549	PAK4	814	TXN

20	ALDH2	285	GNMT	550	PAPSS2	815	TXNIP
21	ALDOA	286	GPC3	551	PARK7	816	TYK2
22	ALKBH3	287	GRIK2	552	PARP1	817	TYMS
23	ALOX15B	288	GRK4	553	PATZ1	818	UBE2C
24	ANAPC1	289	GRK6	554	PAX8	819	UBE2I
25	ANLN	290	GRN	555	PBRM1	820	UBE2N
26	ANXA5	291	GRPR	556	PCGF2	821	UBE2V1
27	APEX1	292	GRSF1	557	PDCD10	822	UBE2V2
28	AR	293	GSK3A	558	PDCD11	823	UBE3A
29	ARF1	294	GSK3B	559	PDCD4	824	UBTD1
30	ARG2	295	GTSE1	560	PDGFB	825	UHRF1
31	ARID1A	296	HAS1	561	PDIK1L	826	ULK3
32	ARID1B	297	HAUS4	562	PDPK1	827	USP1
33	ARID3A	298	HBP1	563	PDZD2	828	USP28
34	ARID4B	299	HDAC1	564	PEA15	829	UTP6
35	ARPC1B	300	HDAC2	565	PEBP1	830	VCAN
36	ARRB1	301	HDAC3	566	PELP1	831	VDR
37	ASAH1	302	HDAC4	567	PES1	832	VEGFA
38	ASF1A	303	HDAC7	568	PEX19	833	VENTX
39	ASPH	304	HELLS	569	PGR	834	WEE1
40	ASXL2	305	HEPACAM	570	PHB	835	WIF1
41	ATF3	306	HIF1A	571	PHB2	836	WIPI1

42	ATF6	307	HIPK2	572	PHGDH	837	WNT16
43	ATF7IP	308	HIRA	573	PI4KB	838	WNT2
44	ATG10	309	HIVEP1	574	PIK3C2A	839	WNT5A
45	ATG12	310	HJURP	575	PIK3CA	840	WNT7B
46	ATG16L1	311	HK3	576	PIK3R5	841	WRN
47	ATG4D	312	HMGA1	577	PIM1	842	WSB1
48	ATG5	313	HMGA2	578	PIN1	843	WT1
49	ATG7	314	HMGB1	579	PINK1	844	WWP1
50	ATM	315	HMGB2	580	PINX1	845	XAF1
51	ATP6V0A2	316	HMGB3	581	PIR	846	XIAP
52	ATP6V0C	317	HMGCR	582	PITX1	847	XPC
53	ATR	318	HNRNPA1	583	PLA2G2A	848	XPO1
54	ATRAID	319	HNRNPA3	584	PLA2R1	849	YAP1
55	ATRX	320	HNRNPAB	585	PLD2	850	YBX1
56	ATXN10	321	HNRNPC	586	PLK1	851	YEATS4
57	AURKA	322	HNRNPD	587	PML	852	YPEL3
58	AURKB	323	HOPX	588	PMVK	853	YWHAB
59	AXL	324	HOXA9	589	PNPT1	854	YWHAZ
60	BAG3	325	HRAS	590	PON1	855	YY1
61	BAP1	326	HS2ST1	591	POT1	856	ZCCHC10
62	BAZ1A	327	HSF1	592	POU3F1	857	ZDHHC3
63	BCL11B	328	HSP90AA1	593	POU5F1	858	ZEB1

64	BCL2	329	HSP90AB1	594	PPARG	859	ZFP36
65	BCL2L1	330	HSPA1A	595	PPARGC1A	860	ZFX
66	BCL2L2	331	HSPA2	596	PPIB	861	ZMAT3
67	BCL3	332	HSPA5	597	PPM1B	862	ZMPSTE24
68	BCL6	333	HSPA9	598	PPM1D	863	ZMYND11
69	BCLAF1	334	HSPB1	599	PPP1R13B	864	ZNF148
70	BECN1	335	HTRA1	600	PPP2R1A	865	ZNF207
71	BHLHE40	336	HYOU1	601	PPP2R5A	866	ZNF217
72	BIN1	337	ID1	602	PRKAA2	867	GHR
73	BIRC5	338	IFI16	603	PRKCD	868	GHRH
74	BLK	339	IFNG	604	PRKCH	869	SHC1
75	BLVRA	340	IGFBP1	605	PRKD1	870	POU1F1
76	BMI1	341	IGFBP3	606	PRKD2	871	PROP1
77	BMP4	342	IGFBP5	607	PRKDC	872	TERC
78	BMPR2	343	IGFBP7	608	PRKN	873	PLAU
79	BMS1	344	IKBKG	609	PRMT1	874	ERCC2
80	BNIP3L	345	IL1A	610	PRMT6	875	ERCC8
81	BRAF	346	IL1R1	611	PRODH	876	IGF1R
82	BRCA1	347	IL1RN	612	PROX1	877	PTPN11
83	BRD4	348	IL24	613	PRPF19	878	NRG1
84	BRD7	349	IL6	614	PSMA2	879	GH1
85	BRIP1	350	ILF3	615	PSMA5	880	IL7R

86	BTG1	351	ILK	616	PSMB1	881	IGF1
87	BTG2	352	IMMT	617	PSMB5	882	IGF2
88	BTG3	353	INCENP	618	PSMD14	883	INS
89	BUB1	354	ING1	619	PTEN	884	NGF
90	BUB1B	355	ING2	620	PTGS2	885	IRS1
91	CALR	356	ING5	621	PTK2	886	IRS2
92	CAPNS1	357	INPP4B	622	PTPN1	887	PIK3CB
93	CARF	358	IRAK4	623	PTPN6	888	NGFR
94	CARM1	359	IRF1	624	PTTG1	889	INSR
95	CASP2	360	IRF3	625	PURB	890	NCOR1
96	CAV1	361	IRF5	626	PYGL	891	JUND
97	CAVIN1	362	IRF7	627	RACGAP1	892	IL2
98	CBS	363	ITGB3	628	RACK1	893	EGF
99	CBX5	364	ITGB4	629	RAD21	894	IL2RG
100	CBX7	365	ITPK1	630	RAD23B	895	PDGFRB
101	CBX8	366	ITPKB	631	RAD51C	896	SST
102	CCL2	367	ITPR1	632	RAF1	897	PPARA
103	CCN2	368	ITPR2	633	RAN	898	RET
104	CCN6	369	ITPR3	634	RANBP9	899	PLCG2
105	CCNA2	370	ITSN2	635	RAP1GAP	900	PEX5
106	CCNB1	371	JAK2	636	RAPGEF4	901	TCF3
107	CCND1	372	JPT1	637	RARB	902	CREBBP

108	CCND3	373	JUN	638	RASSF1	903	UBB
109	CD28	374	JUNB	639	RASSF4	904	RPA1
110	CD34	375	KAT5	640	RASSF5	905	BLM
111	CD40LG	376	KCNA1	641	RB1	906	S100B
112	CD82	377	KCNJ12	642	RBBP4	907	VCP
113	CDC25A	378	KDM1A	643	RBL1	908	POLG
114	CDC45	379	KDM2B	644	RBL2	909	NR3C1
115	CDC6	380	KDM4A	645	RBM38	910	EGR1
116	CDC7	381	KDM4C	646	RBM39	911	ABL1
117	CDCA2	382	KDM5B	647	RBP1	912	BRCA2
118	CDCA4	383	KDM6B	648	RBP2	913	TOP2A
119	CDH1	384	KDR	649	RBPJ	914	TOP2B
120	CDK1	385	KEAP1	650	RBX1	915	NFKB1
121	CDK18	386	KIF11	651	RCC1	916	RAD51
122	CDK2	387	KIF20A	652	RECK	917	TNF
123	CDK2AP1	388	KIF2C	653	RECQL4	918	CEBPA
124	CDK4	389	KIFC1	654	RELA	919	MXI1
125	CDK5	390	KL	655	RELB	920	TGFB1
126	CDK5R1	391	KLF4	656	RHOA	921	ERCC6
127	CDK6	392	KNDC1	657	RNASEH2A	922	STK11
128	CDKN1A	393	KRT19	658	RNASEH2B	923	APTX
129	CDKN1B	394	KSR2	659	RNASEL	924	HTT

130	CDKN1C	395	LAMP2	660	ROMO1	925	PRKCA
131	CDKN2A	396	LATS1	661	RPL11	926	SSTR3
132	CDKN2AIP	397	LATS2	662	RPS14	927	APOC3
133	CDKN2B	398	LAYN	663	RPS6KB1	928	EEF2
134	CEACAM1	399	LBR	664	RPS9	929	ERCC3
135	CEBPB	400	LCN2	665	RPTOR	930	TERF1
136	CEBPG	401	LEO1	666	RRAD	931	CAT
137	CENPA	402	LGALS3	667	RRAS2	932	ERCC5
138	CHAF1B	403	LIMA1	668	RRM1	933	GTF2H2
139	CHD5	404	LIMK1	669	RRM2	934	XRCC5
140	CHEK1	405	LIN52	670	RRM2B	935	PCNA
141	CHEK2	406	LIN54	671	RRP8	936	FEN1
142	CHUK	407	LIN9	672	RTN4	937	FAS
143	CIP2A	408	LMNA	673	RUNX1	938	XRCC6
144	CIT	409	LMNB1	674	RUVBL2	939	POLD1
145	CKAP2	410	LNCTAM34A	675	S100A6	940	BAX
146	CKB	411	LOX	676	SALL1	941	EMD
147	CLCA2	412	LOXL2	677	SAMHD1	942	GRB2
148	CLPP	413	LPAR1	678	SAT2	943	XPA
149	CLSPN	414	LPAR3	679	SDC1	944	MSRA
150	CLU	415	LRRK2	680	SELENBP1	945	COQ7
151	CNOT6	416	LY6D	681	SELENOH	946	CACNA1A

152	CNOT6L	417	MAD1L1	682	SENP1	947	LRP2
153	COX5B	418	MAD2L1	683	SENP7	948	AIFM1
154	CPEB1	419	MAEL	684	SERPINB2	949	UCHL1
155	CPT1C	420	MAF	685	SERPINE1	950	APP
156	CREG1	421	MAGEA2	686	SETD1A	951	APOE
157	CRISPLD2	422	MAGOHB	687	SFN	952	A2M
158	CSNK1A1	423	MAP2K1	688	SFRP1	953	SNCG
159	CSNK2A1	424	MAP2K2	689	SGK1	954	PRDX1
160	CTH	425	MAP2K3	690	SIAH1	955	RGN
161	CTNNAL1	426	MAP2K6	691	SIK1	956	ATP5O
162	CTNNB1	427	MAP2K7	692	SIN3B	957	RAD52
163	CTSB	428	MAP3K14	693	SIRT1	958	TOP3B
164	CTSD	429	MAP3K5	694	SIRT2	959	GPX1
165	CUL4B	430	MAP3K6	695	SIRT3	960	GSR
166	CUX1	431	MAP3K7	696	SIRT6	961	GSS
167	CXCL1	432	MAP4K1	697	SIRT7	962	GSTA4
168	CXCR2	433	MAPK1	698	SIX1	963	GSTP1
169	CYB5R3	434	MAPK12	699	SIX6	964	MT-CO1
170	CYBB	435	MAPK14	700	SKP2	965	HSPD1
171	CYP26A1	436	MAPK9	701	SLC13A3	966	HSPA1B
172	DAO	437	MAPKAPK5	702	SLC16A7	967	PCMT1
173	DDAH2	438	MARCHF5	703	SLC25A5	968	MAPK8

174	DDB1	439	MARCKS	704	SLC31A2	969	PTK2B
175	DDB2	440	MAST1	705	SLC52A1	970	IL7
176	DDIT4	441	MATK	706	SLC5A2	971	MED1
177	DEK	442	MAVS	707	SMAD1	972	MAPK3
178	DEPTOR	443	MCAM	708	SMAD2	973	TAF1
179	DGCR8	444	MCL1	709	SMAD3	974	SDHC
180	DHCR24	445	MCM3AP	710	SMAD5	975	HESX1
181	DHRS2	446	MCM7	711	SMAD6	976	PIK3R1
182	DHX9	447	MCRS1	712	SMARCA2	977	BSCL2
183	DICER1	448	MCU	713	SMARCA4	978	AGPAT2
184	DIDO1	449	MDC1	714	SMARCB1	979	EEF1A1
185	DIRAS3	450	MDH1	715	SMARCD1	980	TFAP2A
186	DKC1	451	MDK	716	SMC1A	981	BDNF
187	DLC1	452	MDM2	717	SMC2	982	CREB1
188	DMTF1	453	MDP1	718	SMG1	983	ATF2
189	DNMT1	454	ME1	719	SMURF2	984	TBP
190	DNMT3A	455	ME2	720	SNAI1	985	HSPA8
191	DNMT3B	456	MECP2	721	SOCS1	986	SIN3A
192	DOT1L	457	MED12	722	SOD1	987	DDIT3
193	DPP4	458	MEF2A	723	SOD2	988	POLA1
194	DPY30	459	MEIS2	724	SORBS2	989	MAPT
195	DTL	460	MEN1	725	SOX1	990	CTGF

196	DUSP1	461	MEOX1	726	SOX2	991	MAX
197	DUSP16	462	MET	727	SOX4	992	MXD1
198	DUSP21	463	METTL14	728	SOX5	993	SUMO1
199	DUSP3	464	MIF	729	SP1	994	H2AFX
200	DUSP6	465	MINK1	730	SPAG9	995	HOXB7
201	DYRK1A	466	MITF	731	SPARC	996	HOXC4
202	E2F1	467	MKRN1	732	SPHK1	997	LEP
203	E2F3	468	MME	733	SPI1	998	LEPR
204	E2F7	469	MMP7	734	SPIN1	999	GHRHR
205	ECT2	470	MMP9	735	SPOP	1000	PSEN1
206	EEF1E1	471	MNX1	736	SRC	1001	DLL3
207	EGFR	472	MOB3A	737	SREBF1	1002	PPP1CA
208	EGLN1	473	MORC3	738	SRF	1003	DBN1
209	EGR2	474	MORF4L1	739	SRSF1	1004	NOG
210	EHF	475	MOS	740	SRSF2	1005	ELN
211	EHMT2	476	MRTFA	741	SRSF3	1006	UCP3
212	EID3	477	MRTFB	742	SSX2	1007	UCP2
213	EIF2AK2	478	MSN	743	STAG2	1008	POLB
214	EIF2AK3	479	MST1	744	STAT1	1009	GCLC
215	EIF4E	480	MT1G	745	STAT3	1010	GCLM
216	EIF4EBP1	481	MTDH	746	STAT5A	1011	BUB3
217	EIF4G2	482	MTHFD2	747	STAT5B	1012	RAE1

218	ELAVL1	48<br>3	MTOR	74<br>8	STAT6	1013	PMCH
219	ELOA	48<br>4	MUC4	74<br>9	STIM1	1014	MLH1
220	ENDOG	48<br>5	MUS81	75<br>0	STK32C	1015	CSNK1E
221	ENG	48<br>6	MVK	75<br>1	STK4	1016	PCK1
222	ENO1	48<br>7	MVP	75<br>2	STK40	1017	ARHGAP1
223	ENTPD7	48<br>8	MXD4	75<br>3	STN1	1018	CDC42
224	EP300	48<br>9	MYBBP1A	75<br>4	STUB1	1019	ARNTL
225	EPAS1	49<br>0	MYBL2	75<br>5	SUMO2	1020	CLOCK
226	EPHA3	49<br>1	MYC	75<br>6	SUMO3	1021	HIC1
227	EPHA5	49<br>2	MYCN	75<br>7	SUPT5H	1022	PAPPA
228	EPOR	49<br>3	MYD88	75<br>8	SUV39H1	1023	ADCY5
229	ERBB2	49<br>4	MYLK	75<br>9	SUZ12	1024	GPX4
230	ERCC1	49<br>5	NACC1	76<br>0	SYK	1025	UCP1
231	ERRFI1	49<br>6	NADK	76<br>1	SYT1	1026	FGF23
232	ERVW-1	49<br>7	NAMPT	76<br>2	SYT7	1027	EFEMP1
233	ESPL1	49<br>8	NANOG	76<br>3	TACC3	1028	ERCC4
234	ESR1	49<br>9	NBN	76<br>4	TAGLN	1029	CETP
235	ESRRB	50<br>0	NBR1	76<br>5	TBK1	1030	AGTR1
236	ETS1	50<br>1	NCAPG2	76<br>6	TBPL1	1031	CISD2
237	ETS2	50<br>2	NCAPH2	76<br>7	TBX2	1032	EPS8
238	ETV6	50<br>3	NDRG1	76<br>8	TBX3	1033	KCNA3
239	EWSR1	50<br>4	NDST2	76<br>9	TEAD1	1034	SLC13A1

240	EZH2	505	NEDD4	770	TEAD4	1035	SOCS2
241	FANCD2	506	NEK1	771	TERF2	1036	TPP2
242	FASN	507	NEK2	772	TERT	1037	NCOR2
243	FASTK	508	NEK4	773	TFDP1	1038	SUN1
244	FBXO22	509	NEK6	774	TFG	1039	BAK1
245	FBXO31	510	NEK9	775	TGFB1I1	1040	IGFBP2
246	FBXO4	511	NF1	776	TGFB2	1041	PYCR1
247	FBXO5	512	NF2	777	TGFBI	1042	TP73
248	FBXW11	513	NFE2L2	778	TGFBR1	1043	CNR1
249	FDPS	514	NFKB2	779	TGFBR2	1044	PDGFRA
250	FERMT1	515	NFKBIA	780	THRB	1045	C1QA
251	FERMT2	516	NHEJ1	781	TIGAR	1046	EIF5A2
252	FGF21	517	NINJ1	782	TIMELESS	1047	DGAT1
253	FGFR1	518	NIPA2	783	TLR10	1048	MT1E
254	FGFR2	519	NLK	784	TLR2	1049	HTRA2
255	FGFR3	520	NLRX1	785	TLR3	1050	NUDT1
256	FIS1	521	NOLC1	786	TLR4	1051	IKBKB
257	FLT1	522	NOTCH1	787	TLR8	1052	SQSTM1
258	FNTB	523	NOTCH3	788	TMEM9B	1053	CDK7
259	FOS	524	NOX1	789	TNFSF13	1054	SPRTN
260	FOXA1	525	NOX4	790	TNFSF15	1055	RICTOR
261	FOXD1	526	NPM1	791	TOM1	1056	CTF1

262	FOXM1	527	NQO1	792	TOP1	1057	TRAP1
263	FOXO1	528	NR1H2	793	TOP3A	1058	TRPV1
264	FOXO3	529	NR2E1	794	TOPBP1	1059	NFE2L1
265	FOXO4	530	NRAS	795	TP53	1060	IFNB1
						1061	GDF11

Supplementary Table S2. Differential expression consistency of core SRGs before and after exclusion of the GSE96971 dataset.

Gene	log2FC (Original)	Adjusted p-value (Original)	log2FC (Excluding GSE96971)	Adjusted p-value (Excluding GSE96971)	Direction	Consistent
SOCS1	-0.550	1.86e-07	-0.550	2.75e-07	Down	Yes
SOCS2	-0.693	1.44e-08	-0.693	1.87e-08	Down	Yes
GADD45G	-0.898	2.84e-08	-0.898	4.25e-08	Down	Yes

Note: Differential expression results were compared before and after exclusion of the GSE96971 dataset. Consistency indicates whether the direction of expression change remained unchanged.

Supplementary Table S3. Diagnostic performance of the three-gene SRG signature in the independent GSE135251 validation cohort.

**Model**	**AUC**	**95% CI**	**Sensitivity**	**Specificity**	**AUPRC**
Three-gene signature	0.936	0.868–0.989	83.01%	90.00%	0.997
SOCS1	0.803	0.675–0.914	47.09%	100.00%	0.988
SOCS2	0.907	0.816–0.971	86.41%	90.00%	0.995
GADD45G	0.911	0.798–0.991	81.55%	90.00%	0.994

Note: Sensitivity and specificity were calculated based on the optimal cutoff value of each signature. AUC, area under the receiver operating characteristic curve; CI, confidence interval; AUPRC, area under the precision–recall curve.

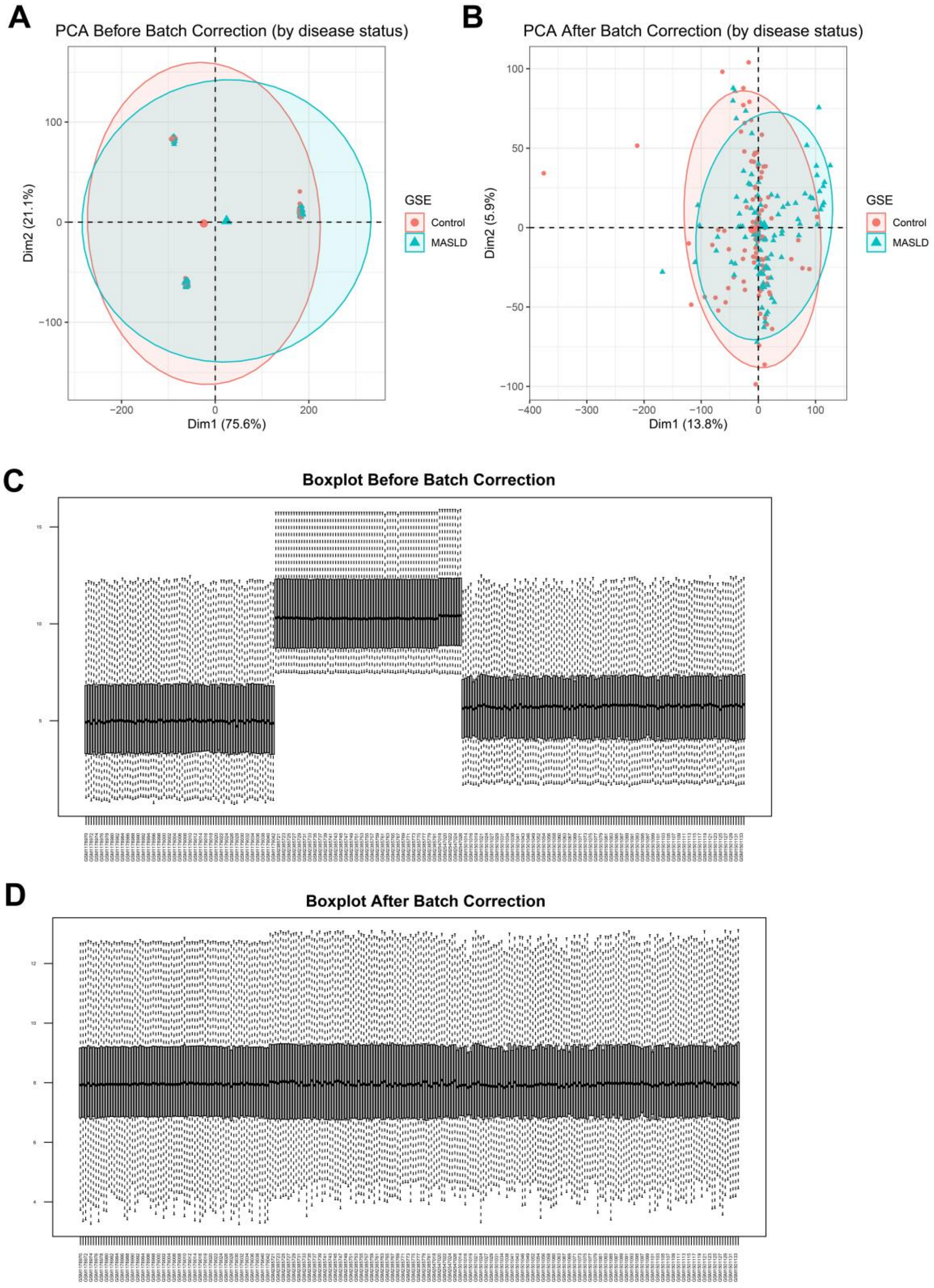

Supplementary Figure S1. Assessment of batch effect correction in integrated GEO datasets.

(A–B) PCA plots before (A) and after (B) batch correction showing reduced technical

variation while preserving biological differences between MASLD and control samples.

(C–D) Boxplots showing the distribution of gene expression values before (C) and after (D) batch correction, demonstrating improved consistency across datasets.

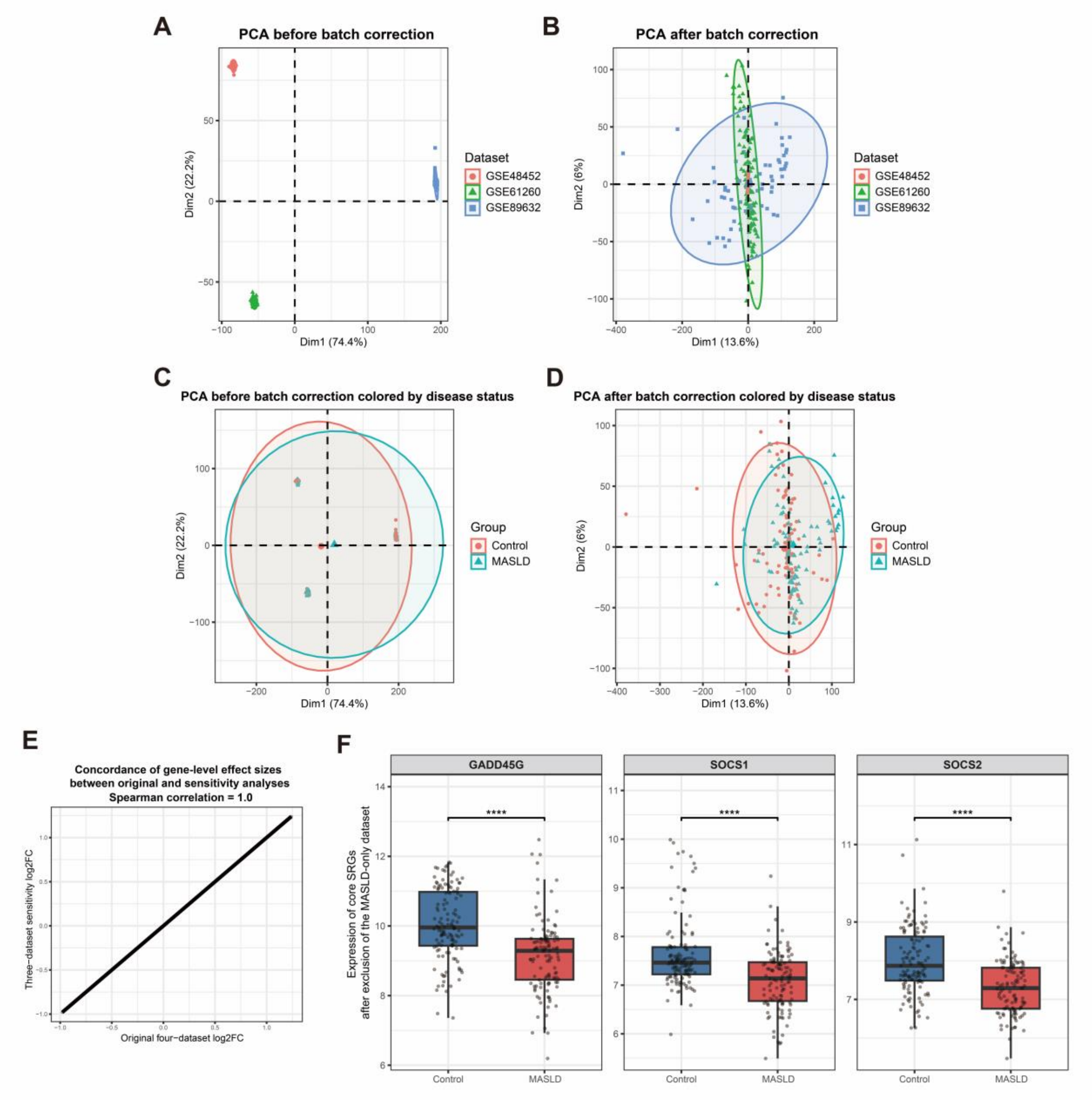

Supplementary Figure 2. Sensitivity analysis demonstrates the robustness of transcriptomic integration after exclusion of the MASLD-only dataset.

(A-B) PCA plots showing dataset distribution before and after batch correction. Batch-associated variation was reduced after correction.

(C-D) PCA plots colored by disease status showing preservation of MASLD-associated transcriptional patterns after batch correction.

(E) Correlation analysis of gene-level log2 fold changes between the original four-dataset analysis and sensitivity analysis excluding the MASLD-only (GSE96971)

dataset.

(F) Expression patterns of GADD45G, SOCS1, and SOCS2 remained consistent after exclusion of the MASLD-only dataset.

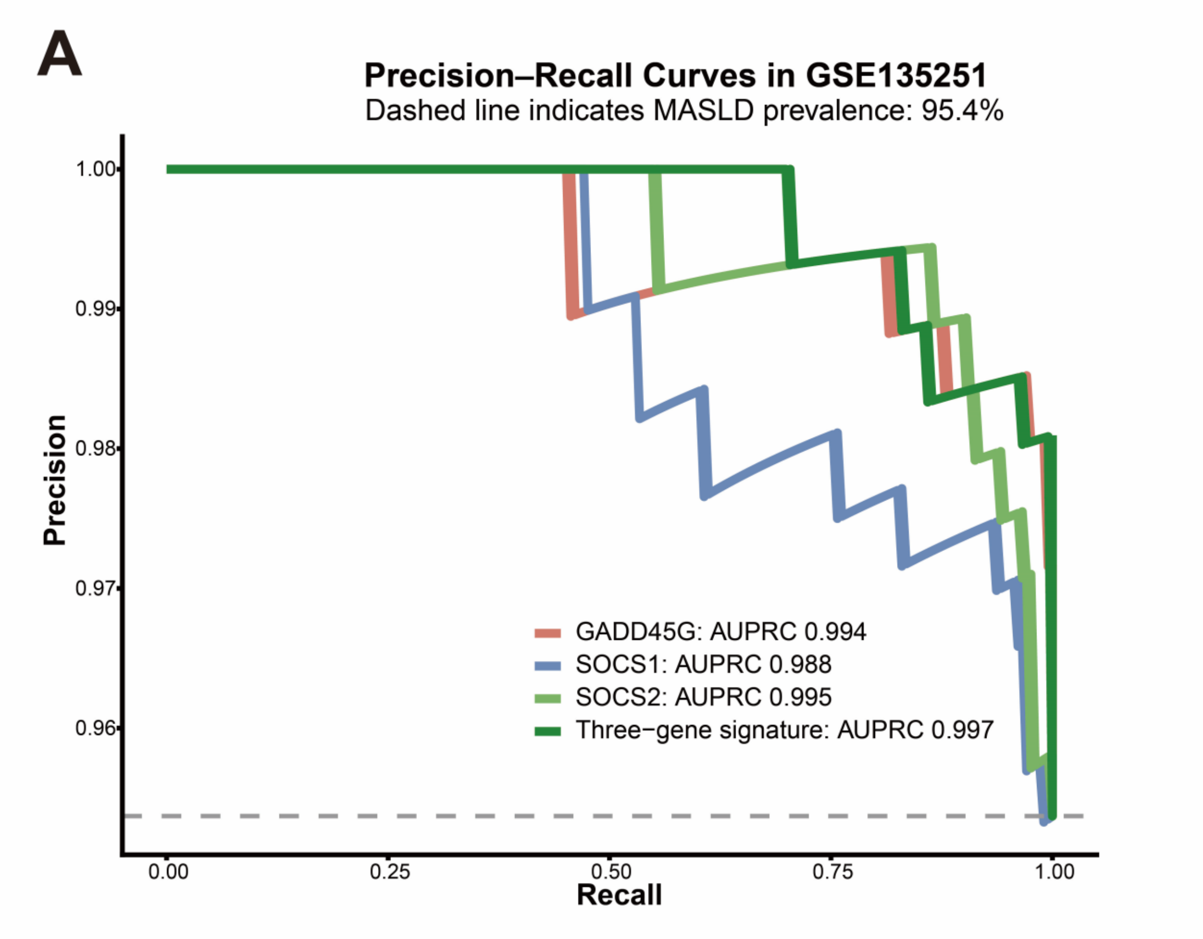

Supplementary Figure S3. Precision–recall curve analysis of the SRG signature in the GSE135251 cohort.

(A) Precision–recall curves demonstrated robust classification performance of the three-gene signature and individual SRGs in the GSE135251 cohort. The AUPRC values were 0.997 for the three-gene signature, 0.988 for SOCS1, 0.995 for SOCS2, and 0.994 for GADD45G.

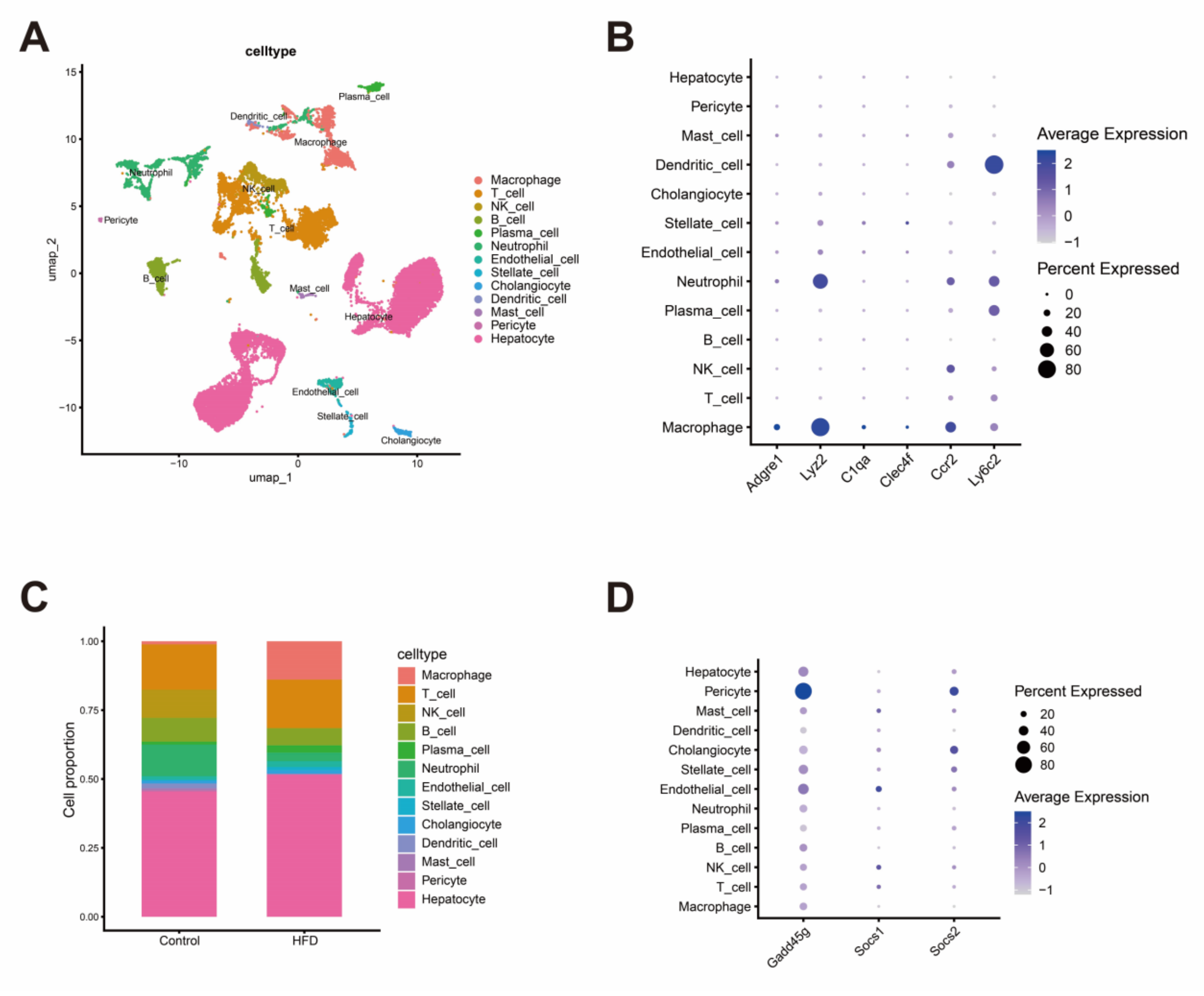

Supplementary Figure S4. Single-cell RNA sequencing analysis of Gadd45g expression patterns in mouse liver.

(A) UMAP visualization of major liver cell populations identified from the

GSE300744 single-cell RNA sequencing dataset. Distinct cell clusters were annotated based on canonical marker genes.

(B) Dot plot showing the expression of representative marker genes used for cell-type annotation, including Adgre1, Lyz2, C1qa, Clec4f, Ccr2, and Ly6c2.

(C) Cell composition analysis showing the distribution of major liver cell populations in control and HFD-fed mice.

(D) Dot plot showing the expression patterns of Gadd45g, Socs1, and Socs2 across different liver cell populations. Gadd45g expression was detected in multiple cell types and showed a macrophage-associated expression pattern.
